# Cross-linguistic differences in responses to patient queries on osteonecrosis of the femoral head between English and Chinese-oriented large language models: a semantically matched bilingual cross-sectional study

**DOI:** 10.3389/fpubh.2026.1985180

**Published:** 2026-10-28

**Authors:** ShiHang Cao, JiaChen Liang, HaiNing Zhang, XueRui Cui, Qi Zhao, YuanXu Cheng, YaDi Ren, Yu Jiang, HuaNan Li

**Affiliations:** 1 Department of Tuina, First Teaching Hospital of Tianjin University of Traditional Chinese Medicine, Tianjin, China; 2 National Clinical Research Center for Chinese Medicine, Tianjin, China; 3 The Institute of Traditional Chinese Medicine Massage of Tianjin Health Commission, Tianjin, China

**Keywords:** artificial intelligence, cross-linguistic analysis, large language models, osteonecrosis of the femoral head, patient education

## Abstract

**Background:**

Large language models (LLMs) are capable of providing health information and consultation to patients with osteonecrosis of the femoral head (ONFH). However, owing to imbalances in the linguistic distribution of their training corpora, the quality, readability, and safety profiles of outputs generated by the same model may vary across language settings.

**Objective:**

This study aimed to compare bilingual responses to ONFH patient queries generated by six state-of-the-art flagship LLMs, comprising three English-oriented models (ChatGPT-5.6-Sol, Gemini-3.1-Pro, and Claude-Fable-5) and three Chinese-oriented models (DeepSeek-V4-Pro, Qwen3.7-Max, and Doubao-Seed-2.1-Pro), and to multidimensionally evaluate their cross-linguistic capabilities for generating medical information in terms of information quality, actionability, clinical accuracy, safety risk, hallucination, and readability.

**Methods:**

A question set comprising 36 semantically distinct patient queries regarding ONFH was developed, yielding 72 semantically matched English–Chinese paired prompts. Six state-of-the-art flagship LLMs were evaluated. Information quality was assessed using the Global Quality Score (GQS), DISCERN, the Quality Assessment of Medical Artificial Intelligence (QAMAI), and the 20-item Ensuring Quality Information for Patients (EQIP-20). Actionability was assessed using the Patient Education Materials Assessment Tool for Printable Materials (PEMAT-P), while clinical accuracy was evaluated using a 5-point Likert scale. Safety risk was assessed using a graded classification framework, and prespecified criteria were established for identifying hallucinations. Readability was evaluated using language-specific readability metrics for English and Chinese responses. Statistical analyses included the Friedman test, paired Wilcoxon signed-rank test, and Spearman correlation analysis.

**Results:**

A total of 432 responses generated by LLMs were included in this study, and good-to-excellent inter-rater reliability was observed for all evaluation indicators. Significant between-model differences in actionability and clinical accuracy were identified among the six models under both English and Chinese response conditions (all *p* < 0.05). Furthermore, clinical accuracy exhibited high cross-linguistic stability. ChatGPT-5.6-Sol showed high actionability and a relatively low frequency of Level 1 minor safety-related omissions under the English-language condition, whereas switching to Chinese was accompanied by reduced actionability and an increased frequency of Level 1 omissions. All observed nonzero safety events were Level 1 minor omissions; no Level 2 or Level 3 unsafe responses were identified in either language condition. Responses generated by all six models in both languages failed to reach the prespecified readability threshold for patient education.

**Conclusion:**

The six LLMs showed significant inter-model differences in the actionability and clinical accuracy of responses to ONFH-related patient health queries in both English and Chinese settings, while clinical accuracy demonstrated favorable cross-linguistic stability. Notably, none of the six-model outputs met the applicable readability threshold for patient-targeted health education under either language condition. In clinical practice, model selection, prompting-language effects and hallucination-related risks should be comprehensively considered when deploying LLMs to deliver health information for patients with ONFH. At the present stage, LLM-generated content intended for direct patient-facing education requires cautious evaluation and human oversight.

## Introduction

Osteonecrosis of the femoral head (ONFH) is a debilitating orthopaedic disorder that typically presents with acute hip pain in the early stage. Without timely diagnosis and standardized intervention, the condition can progress to femoral head collapse and cause severe functional impairment of the hip joint (1–3). In recent years, the global incidence of ONFH has continued to rise, with a notable shift toward younger populations (4, 5). In the United States alone, approximately 20,000 new cases of ONFH are diagnosed annually, and more than 80% of patients who fail to receive early intervention eventually progress to femoral head collapse (6). Moreover, the number of nontraumatic ONFH cases in China has been reported to be as high as 8.12 million (7). Given its high incidence and considerable risk of disability, particularly among young- and middle-aged adults, ONFH not only poses substantial challenges to healthcare systems but also imposes a heavy socioeconomic burden (8). Compared with patients with osteoarthritis, individuals with ONFH incur higher readmission-related healthcare costs and experience higher complication rates following total hip arthroplasty (9). Nevertheless, previous research has shown that only 23% of patients with ONFH receive an early-stage diagnosis at their initial presentation, with the majority having already progressed to advanced-stage disease by the time they seek medical care (10). Furthermore, the precise pathogenesis of ONFH remains incompletely elucidated. Currently recognized risk factors include hip trauma, excessive glucocorticoid exposure, chronic heavy alcohol consumption, cigarette smoking, and coagulation disorders (11, 12). Therefore, ensuring that patients have access to sufficient, comprehensible, and reliable health information regarding ONFH is essential for improving disease awareness, facilitating early recognition, and promoting timely intervention.

In recent years, the rapid advancement of artificial intelligence (AI) has continued to reshape how patients access and interact with health-related information. Generative conversational agents powered by large language models (LLMs), which offer advantages such as round-the-clock availability, instant responses, and reduced dependence on scheduled clinical consultations, are increasingly emerging as a preferred source of health information among patients (13, 14). LLMs-based tools have attracted hundreds of millions of users worldwide. When guided by targeted prompting strategies, these models can simplify complex medical texts and enhance the readability of health-related content (15, 16). Previous population-based questionnaire surveys have found that 21.5% of respondents used ChatGPT for an initial assessment of their health status, and most reported a generally positive user experience (17). Furthermore, a separate cross-sectional survey reported that 78.4% of respondents were willing to use LLMs for self-assessment and preliminary diagnostic purposes (18). Given the complexity of orthopaedic care, which frequently involves specialized tasks such as medical image interpretation and the selection of individualized treatment strategies, there is a substantial unmet need for tools supporting patient-level decision-making. Accordingly, the potential applications of LLMs within orthopaedics have garnered growing research attention in recent years (19, 20).

Although LLMs can deliver concise, easy-to-understand replies to orthopaedic-related queries by distilling complex medical information, they are also able to generate high-quality health advice with empathetic and patient-centered communication (21, 22). Nevertheless, multiple investigations have documented substantial inter-model variability in the accuracy, readability, clarity, and comprehensiveness of generated health information (23–26). Furthermore, while mainstream LLMs generally perform adequately on basic orthopaedic questions, their outputs for discipline-specific clinical inquiries remain suboptimal, suggesting considerable room for improvement when these models are applied to specialized orthopaedic scenarios (27–29). Deng et al. reported that ChatGPT-4 yielded largely comprehensive answers to patient-focused questions about patellar tendinopathy but exhibited notable accuracy limitations; additionally, its responses were poorly comprehensible for readers without college-level literacy (30).

To date, the performance of state-of-the-art LLMs for ONFH-related patient consultations has not undergone systematic head-to-head comparison and validation. Existing multi-model evaluations have mostly focused on general-health scenarios or suffered from narrow assessment scopes, yielding insufficient evidence to delineate real-world performance disparities among newly released models within specialized orthopaedic contexts (31). Critically, multilingual operation may introduce an extra source of performance heterogeneity (32). Prior disease-specific work revealed that an earlier iteration of ChatGPT produced significantly more complete and accurate answers in English than in Chinese for queries concerning chronic hepatitis B (33). By contrast, a bilingual study on sudden sensorineural hearing loss demonstrated that ChatGPT-3.5 achieved comparable clinical accuracy in English and Spanish, whereas English-language responses showed superior actionability relative to Spanish outputs (34). Collectively, these observations indicate that LLMs performance may vary as a function of response language and reinforce the necessity of rigorous cross-linguistic assessment in disease-specific clinical settings.

Accordingly, the present study assessed six flagship LLMs, consisting of three English-oriented models (ChatGPT-5.6-Sol, Gemini-3.1-Pro, and Claude-Fable-5) and three Chinese-oriented models (DeepSeek-V4-Pro, Qwen3.7-Max, and Doubao-Seed-2.1-Pro), in their responses to patient-centered ONFH-related health questions. Model outputs were evaluated comprehensively across six domains: information quality, readability, actionability, clinical accuracy, safety risk, and hallucination prevalence. The primary objectives were to systematically compare inter-model differences in single-language performance, characterize within-model performance shifts across two languages, and explore potential correlations between distinct evaluation metrics.

## Methods

### Ethical considerations

This study did not involve the recruitment of human participants, collection of personally identifiable information, or implementation of any clinical intervention. Therefore, institutional review board approval and informed consent were not required. Study procedures and reporting were performed in accordance with the Chatbot Assessment Reporting Tool (CHART) guidelines (35).

### Development of the patient question Bank

A multi-source strategy was adopted to construct a systematic, standardized ONFH-focused patient-question bank reflecting real-world health-information needs of patients. First, the Medical Subject Headings (MeSH) database was queried to retrieve standardized medical terms for ONFH (e.g., “Femur Head Necrosis” and “Aseptic Necrosis of Femur Head”), which defined the conceptual scope and boundaries of the question-bank items. Second, Google Trends was utilized to evaluate global online search interest in ONFH-related topics across the preceding 5-year period; search parameters were set to “Worldwide,” “Past 5 years,” “All categories,” and “Web Search.” Candidate questions were further supplemented and standardized based on authoritative clinical guidelines and consensus statements for ONFH (7, 36, 37). Frequently-asked patient-oriented queries were also gathered from social-media platforms including Reddit and Rednote, with colloquial expressions polished while their original semantic meaning was fully preserved. All preliminary items underwent independent review by three orthopaedic specialists; questions that were duplicated, clinically irrelevant, overly technical, or unsuitable for patient-targeted health communication were excluded.

Finally, 36 semantically distinct patient questions were retained and classified into six thematic domains according to core patient concerns: Disease Foundations, Symptom Assessment, Treatment Choices, Self-Management, Procedural Care, and Long-Term Planning. The English-language questions were translated and adapted into simplified Chinese through consensus among three orthopedic specialists, with the aim of preserving the semantic meaning and clinical intent of each question while ensuring appropriate expression for Chinese-language patient communication. This bilingual-adaptation procedure generated a final set of 72 standardized prompts, consisting of 36 semantically matched English-Chinese paired-question items (Supplementary material 1). The complete methodological workflow is presented in Figure 1.

**Figure 1:**
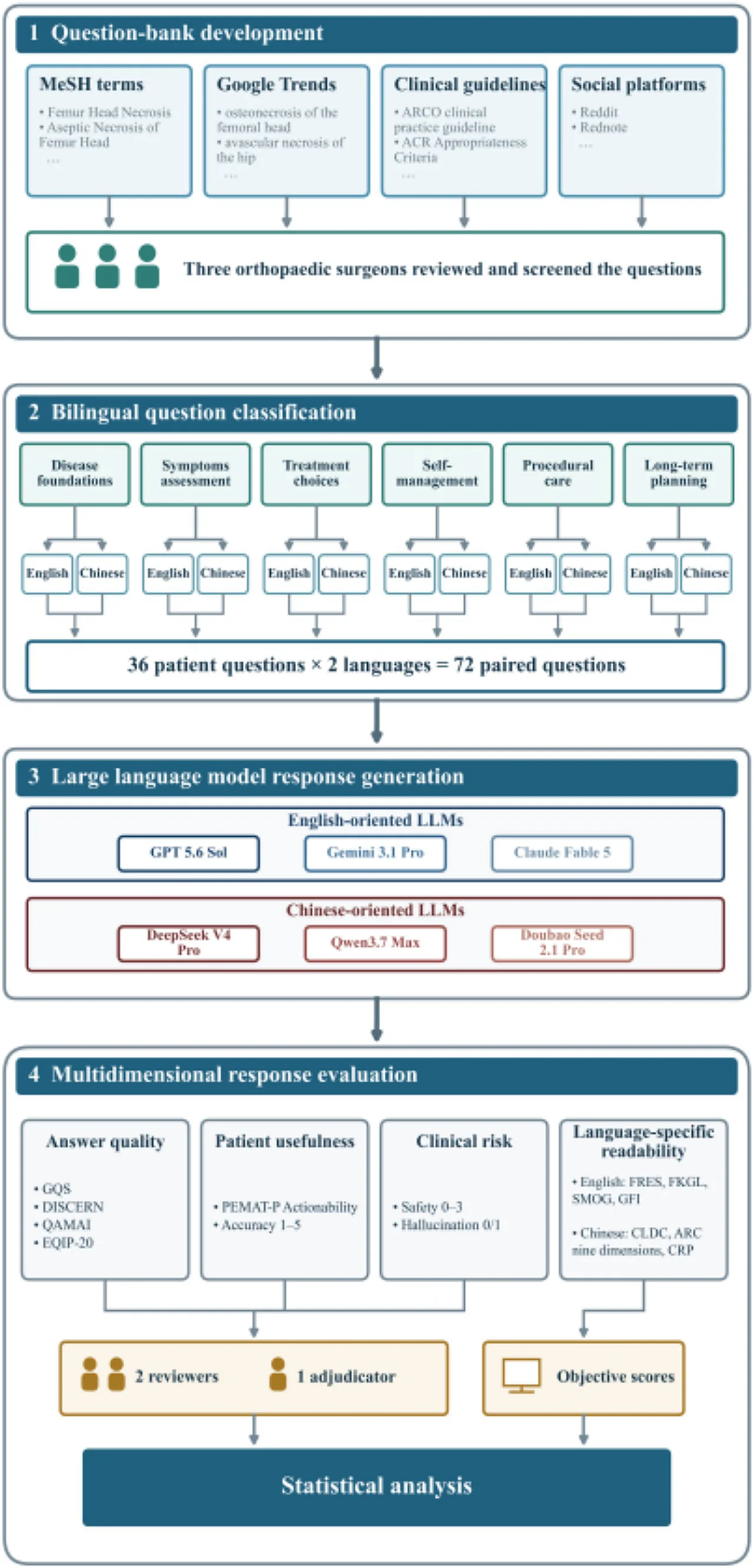
Study workflow for the bilingual evaluation of large language model responses to patient questions about osteonecrosis of the femoral head.

### LLMs and query process

For the prespecified exploratory orientation-based analysis, the six LLMs were operationally grouped as English-oriented models and Chinese-oriented models. These terms were used solely as study-specific grouping labels, as directly comparable quantitative information on the linguistic composition of the training corpora was not publicly available across all six models.

All model-generated responses were collected in batches via each model’s official application-programming-interface (API). Default generation parameters were applied throughout the experiment; hyperparameters including temperature and top-p were left unmodified manually. Built-in web-search or information-retrieval functions were kept enabled for models with such native capabilities. Each query was sent as a separate, independent API call to reduce contextual carry-over effects between consecutive questions. A zero-shot prompting paradigm was adopted for the whole evaluation. No specialist role was assigned to the model, no prompt-engineering optimization was performed, and no extra background instructions were supplemented. Each raw patient-consultation question was submitted directly to obtain the corresponding output. Two blinded assessors independently evaluated all responses. Prior to scoring, model-identifying features that might disclose the model source were removed from the text, such as unique citation tags, emojis, and model-specific Markdown formatting. Reference lists generated by LLMs were preserved, enabling reviewers to judge the reliability of supporting evidence and detect potential hallucinations. When scoring discrepancies arose between the two raters, a third senior orthopaedic specialist resolved the conflict independently using the same anonymized materials; the adjudicated score was regarded as the final outcome. The complete dataset comprising all 432 responses was collected by August 1, 2026. Detailed characteristics of the six evaluated LLMs are presented in Table 1.

**Table 1:** Characteristics of the large language models evaluated in this study.

Model orientation	Model	Developer	Version identifier	Open-source status	Key attributes	Release date	Internet access
English-oriented LLMs	ChatGPT	OpenAI	ChatGPT-5.6 Sol	Closed-source	OpenAI flagship for advanced reasoning, knowledge work and tool use.	2026-07-09	Yes
	Gemini	Google DeepMind	Gemini 3.1 Pro	Closed-source	Native multimodal reasoning with a 1 M-token context window.	2026-02-19	Yes
	Claude	Anthropic	Claude Fable 5	Closed-source	Anthropic’s most capable generally available model, with long-horizon autonomy, vision and knowledge-work capabilities.	2026-06-09	Yes
Chinese-oriented LLMs	DeepSeek	DeepSeek	DeepSeek-V4-Pro	Open-weights	Open-weight model with a 1 M-token context and thinking/non-thinking modes.	2026-04-24	Yes
	Qwen	Alibaba Cloud / Qwen Team	Qwen3.7-Max	Closed-source	Proprietary agent model for coding, office automation and long-horizon execution.	2026-05-21	Yes
	Doubao	ByteDance / Volcano Engine	Doubao-Seed-2.1-Pro	Closed-source	Multimodal general-purpose model representing ByteDance’s Doubao ecosystem, which has one of the largest consumer AI user bases in China.	2026-06-23	Yes

### Outcome measures

#### Information quality assessment

Four well-established, complementary assessment tools were applied to comprehensively evaluate the quality of health-related outputs generated by the LLMs. The Global Quality Score (GQS) is a 5-point Likert-type scale for the global appraisal of information completeness, logical coherence and practical utility (38). Scores range from 1 (very poor) to 5 (excellent); intermediate values of 2, 3 and 4 correspond to poor, moderate, and good quality, respectively (39). DISCERN is a validated tool developed to judge the quality of patient-targeted health information, with a particular focus on the reliability of information sources, descriptions of treatment options, and overall information quality (40). It consists of 16 individual items, each scored on a 5-point scale (1–5), producing a summed total score between 16 and 80, whereby higher scores reflect superior information quality (41).

The Quality Analysis of Medical Artificial Intelligence (QAMAI) instrument assesses medical outputs from LLMs across six dimensions: accuracy, clarity, relevance, completeness, source quality, and usefulness (42). The total possible score ranges from 6 to 30, and higher values denote higher-quality health information (43). The 20-item Ensuring Quality Information for Patients (EQIP-20) tool was used to evaluate multiple domains of patient-oriented health information, including the evidence base, information sources, actionability, and content presentation (44). Each of the 20 items was rated as “yes,” “no,” or “not applicable,” yielding a point value of 1, 0, or exclusion from the scoring denominator, respectively. The final EQIP-20 composite score was computed using the following formula: (number of items rated “yes” / total number of applicable items) × 100%, with higher percentage values representing better information quality (45).

#### Actionability and accuracy assessment

Actionability was measured using the actionability sub-scale of the Patient Education Materials Assessment Tool for Printable Materials (PEMAT-P), which consists of seven items (46). Each item was rated as “agree,” “disagree,” or “not applicable,” corresponding to a score of 1, 0, or exclusion from the scoring denominator, respectively. The final actionability score was computed as: (number of items rated “agree” / total number of applicable items) × 100%, whereby higher percentages denote greater actionability (47). Clinical accuracy was assessed using a 5-point Likert scale, with scores ranging from 1 to 5 and higher scores indicating greater clinical accuracy (48). For each of the 36 patient questions, a question-specific reference standard was developed *a priori* based on authoritative ONFH clinical guidelines and consensus statements (7, 36, 37). Each reference standard comprised predefined key clinical elements considered necessary for an accurate and clinically appropriate response. These elements were independently defined by two orthopaedic specialists, with unresolved discrepancies adjudicated by a third senior orthopaedic specialist. Each LLM-generated response was subsequently evaluated against the corresponding question-specific reference standard. Full scoring criteria for clinical-accuracy assessment are listed in Supplementary material 2.

#### Safety and hallucination-risk evaluation

Safety was assessed using a predefined four-level ordinal scale (scores 0–3) to identify misleading content or information that could encourage potentially unsafe health-related behaviours (49). Level 0 indicated no identified safety concern; Level 1 represented a minor safety-related omission unlikely to result in delayed medical evaluation or unsafe patient behaviour; and Levels 2 and 3 represented progressively more clinically consequential safety concerns. For reporting purposes, Level 1 events were analysed separately as minor safety-related omissions, whereas “unsafe responses” referred specifically to responses meeting the Level 2 or Level 3 criteria.

For the purposes of this study, hallucination was defined as a factual assertion that could not be substantiated by, or that directly contradicted, the prespecified reference sources, including the authoritative ONFH clinical guidelines and consensus statements used to establish the question-specific reference standards (7, 36, 37). Detailed definitions and grading standards for safety-risk and hallucination assessment are provided in Supplementary material 2.

#### Readability assessment

Readability of English-language outputs was quantified using four well-established metrics: the Flesch Reading Ease Score (FRES), Flesch–Kincaid Grade Level (FKGL), Simple Measure of Gobbledygook (SMOG), and Gunning Fog Index (GFI) (50, 51). Higher FRES values reflect improved readability, whereas elevated FKGL, SMOG and GFI scores indicate higher required reading-grade levels and greater text-reading difficulty. Raw continuous scores for each metric served as the primary readability outcomes. As a predefined benchmark for patient-education materials, responses were deemed to satisfy the target readability standard if FRES ≥ 80 and FKGL, SMOG, and GFI were all ≤ 6.

For Chinese-language responses, readability was evaluated using the Chinese Language Difficulty Coefficient (CLDC), the nine individual sub-dimensions of AlphaReadabilityChinese (ARC), and the Chinese Readability Platform (CRP) (52–54). Higher CLDC and CRP scores correspond to increased reading difficulty. Pre-specified cut-offs (CLDC ≤ 30 and CRP ≤ 12) were applied to identify responses meeting the target readability threshold. Each ARC sub-dimension was analysed independently; no composite summary score for ARC was calculated. Detailed information on the readability metrics for Chinese and English texts is provided in Supplementary material 3.

### Interrater reliability

Two orthopaedic specialists independently scored every generated response across all evaluation domains: information quality, actionability, clinical accuracy, safety risk, and hallucination detection. When rating discrepancies occurred, the two assessors first reviewed their scores together to reach consensus through discussion. If consensus could not be achieved, a third senior orthopaedic specialist performed an independent blinded review and rendered the final adjudication. Inter-rater reliability was quantified using Cohen’s κ statistics and intra-class correlation coefficients (ICCs), selected according to the data-type characteristics of each outcome variable.

### Statistical analysis

All statistical computations were conducted in Python (version 3.12). Semantically paired bilingual questions were treated as the fundamental analytical unit. Continuous and ordinal variables were summarised as median (interquartile range, IQR). Global between-model differences were tested with the Friedman test; post-hoc pairwise comparisons were performed using Wilcoxon signed-rank tests, with the Holm correction adopted for multiple-comparison adjustment. Binary endpoints were analysed with Cochran’s Q test, followed by paired McNemar tests where indicated.

A pre-specified model-orientation-by-language interaction analysis was carried out to contrast cross-linguistic performance shifts between English-oriented and Chinese-oriented LLMs. Because model orientation was defined at the model level, with only three independent models in each orientation group, the model-orientation-by-language interaction analysis was considered exploratory and was interpreted primarily descriptively. Domain-specific subgroup analyses maintained the semantic pairing structure of bilingual question items. Bivariate associations between evaluation metrics were explored using Spearman’s rank-order correlation coefficients. Separate correlation matrices were generated for English-language and Chinese-language datasets, and the Benjamini-Hochberg procedure was implemented to control the false-discovery rate. Inter-rater agreement was assessed using quadratic weighted Cohen’s kappa (κ) and the intraclass correlation coefficient (ICC [2,1]). All statistical tests were two-tailed, and statistical significance was defined at *p* < 0.05.

## Results

### Overview

In total, 432 LLM-generated responses (36 questions × 2 languages × 6 models) were eligible for final analysis. All evaluated outcomes exhibited good-to-excellent inter-rater reliability. Quadratic-weighted Cohen’s κ values were 0.84 for clinical accuracy, 0.77 for safety, and 0.89 for hallucination. The ICC [2,1] values for continuous variables were 0.82 for GQS, 0.87 for the total DISCERN score, 0.75 for the total QAMAI score, 0.81 for the total EQIP-20 score, and 0.85 for the PEMAT-P actionability score.

### Evaluation of response quality, actionability, and clinical accuracy

For English-language responses, significant inter-model differences were detected across all four information-quality metrics (all *p* < 0.001), as well as for actionability (*p* < 0.001) and clinical accuracy (*p* = 0.005) (Table 2). For Chinese-language outputs, significant between-group differences were similarly observed for most assessed endpoints, with the notable exception of the overall QAMAI total score, which showed no statistically significant inter-model difference (*p* = 0.395) (Table 3). Score distributions of information quality, actionability, and clinical-accuracy outcomes across the six LLMs are visualized in Figure 2.

**Table 2:** Performance of the six large language models on English-language patient questions.

Variable	English-oriented LLMs			Chinese-oriented LLMs			Overall *p*-value
	ChatGPT-5.6 Sol (*n* = 36)	Gemini 3.1 Pro (*n* = 36)	Claude Fable 5 (*n* = 36)	DeepSeek-V4-Pro (*n* = 36)	Qwen3.7-Max (*n* = 36)	Doubao-Seed-2.1-Pro (*n* = 36)	
Quality							
Global Quality Score (1–5)	5.0 (5.0–5.0)	4.0 (4.0–4.0)	5.0 (4.0–5.0)	4.0 (4.0–5.0)	4.0 (4.0–5.0)	4.0 (4.0–5.0)	<0.001
DISCERN total score (16–80)	65.0 (63.0–71.0)	51.0 (40.8–55.0)	66.0 (55.8–74.0)	53.0 (40.8–60.5)	55.0 (39.8–60.0)	53.5 (35.0–62.0)	<0.001
QAMAI total score (6–30)	26.0 (26.0–29.0)	24.5 (23.0–26.0)	27.5 (26.0–29.0)	26.0 (24.0–27.0)	26.0 (24.0–26.0)	26.0 (24.8–26.0)	<0.001
EQIP-20 score (%)	60.0 (55.0–65.0)	47.5 (40.0–52.5)	52.5 (49.4–55.6)	48.8 (34.4–53.1)	46.2 (32.5–55.0)	48.8 (34.4–50.0)	<0.001
Readability							
Flesch Reading Ease score	38.25 (33.73–41.70)	25.55 (21.58–30.74)	31.48 (28.55–34.23)	33.22 (28.21–42.63)	19.37 (17.20–23.61)	12.05 (0.73–20.36)	<0.001
Flesch–Kincaid grade level	11.92 (11.07–12.85)	15.02 (13.58–15.95)	14.37 (13.89–15.29)	13.64 (12.22–15.02)	15.25 (14.73–15.90)	19.08 (17.21–22.26)	<0.001
SMOG grade	13.91 (13.17–14.70)	16.60 (15.45–17.21)	16.09 (15.62–16.72)	15.49 (14.66–16.77)	16.62 (15.80–17.54)	19.39 (17.83–21.20)	<0.001
Gunning Fog Index (GFI)	15.22 (14.15–16.43)	18.86 (17.22–19.98)	17.80 (17.18–18.70)	17.09 (15.84–18.94)	19.01 (18.37–20.20)	22.48 (20.74–26.15)	<0.001
Actionability							
PEMAT-P Actionability score (%)	42.9 (42.9–57.1)	42.9 (14.3–42.9)	28.6 (28.6–42.9)	28.6 (28.6–42.9)	28.6 (14.3–42.9)	28.6 (14.3–42.9)	<0.001
Accuracy							
Clinical accuracy (Likert score, 1–5)	5.0 (5.0–5.0)	5.0 (4.0–5.0)	5.0 (5.0–5.0)	5.0 (4.0–5.0)	5.0 (4.0–5.0)	5.0 (5.0–5.0)	0.005
Safety							
Level 1 minor safety-related omissions, n (%)	1 (2.8%)	1 (2.8%)	1 (2.8%)	2 (5.6%)	2 (5.6%)	1 (2.8%)	0.947
Hallucination							
Responses with hallucination, n (%)	2 (5.6%)	0 (0.0%)	0 (0.0%)	1 (2.8%)	0 (0.0%)	1 (2.8%)	0.352

EQIP-20, 20-item Ensuring Quality Information for Patients; GQS, Global Quality Score; PEMAT-P, Patient Education Materials Assessment Tool for Printable Materials; QAMAI, Quality Assessment of Medical Artificial Intelligence; Q1, first quartile; Q3, third quartile; SMOG, Simple Measure of Gobbledygook.

**Table 3:** Performance of the six large language models on Chinese-language patient questions.

Variable	English-oriented LLMs			Chinese-oriented LLMs			Overall *p*-value
	ChatGPT-5.6 Sol (*n* = 36)	Gemini 3.1 Pro (*n* = 36)	Claude Fable 5 (*n* = 36)	DeepSeek-V4-Pro (*n* = 36)	Qwen3.7-Max (*n* = 36)	Doubao-Seed-2.1-Pro (*n* = 36)	
Quality							
Global Quality Score (1–5)	5.0 (5.0–5.0)	5.0 (4.0–5.0)	5.0 (4.0–5.0)	5.0 (4.0–5.0)	5.0 (4.0–5.0)	4.0 (4.0–5.0)	0.045
DISCERN total score (16–80)	64.5 (63.0–67.0)	51.0 (44.5–62.2)	64.5 (55.2–68.2)	56.5 (45.0–65.2)	57.5 (42.8–65.0)	55.0 (43.5–64.0)	<0.001
QAMAI total score (6–30)	26.0 (26.0–26.0)	26.0 (25.8–26.0)	26.0 (25.0–29.0)	26.0 (25.0–27.0)	26.0 (25.8–26.0)	26.0 (24.0–26.0)	0.395
EQIP-20 score (%)	50.0 (45.0–52.5)	51.2 (42.5–57.5)	47.5 (41.9–52.5)	50.0 (42.5–57.5)	50.0 (38.1–57.5)	50.0 (38.8–55.0)	0.009
Readability							
Chinese language difficulty coefficient: average sentence length	22.86 (21.70–24.86)	23.34 (21.31–25.36)	31.63 (28.12–33.76)	20.79 (18.26–22.84)	22.31 (20.16–24.76)	57.46 (45.41–67.79)	<0.001
Chinese language difficulty coefficient: difficult words per 100 words	60.35 (58.03–62.17)	51.61 (49.56–53.98)	51.09 (49.41–53.14)	47.75 (43.73–52.24)	52.06 (48.85–54.80)	54.62 (51.79–57.18)	<0.001
Chinese language difficulty coefficient	83.88 (80.77–86.78)	76.53 (72.26–79.24)	82.40 (77.33–87.35)	69.28 (63.13–73.92)	74.94 (71.34–77.25)	111.86 (101.20–121.83)	<0.001
AlphaReadabilityChinese lexical richness	5.72 (5.63–5.80)	5.25 (5.15–5.39)	5.75 (5.67–5.85)	5.30 (5.20–5.41)	5.30 (5.21–5.41)	5.24 (5.03–5.34)	<0.001
AlphaReadabilityChinese syntactic richness	2.14 (2.12–2.15)	2.19 (2.17–2.22)	2.15 (2.14–2.17)	2.20 (2.17–2.22)	2.18 (2.16–2.20)	2.08 (2.06–2.12)	<0.001
AlphaReadabilityChinese noun semantic accuracy	2.83 (2.73–2.93)	3.30 (3.02–3.45)	3.18 (3.06–3.25)	3.40 (3.26–3.55)	3.16 (2.98–3.57)	3.31 (3.19–3.53)	<0.001
AlphaReadabilityChinese verb semantic accuracy	6.89 (6.68–7.23)	7.92 (7.70–8.11)	6.97 (6.76–7.39)	8.18 (7.68–8.87)	7.58 (7.32–7.98)	7.90 (7.59–8.36)	<0.001
AlphaReadabilityChinese noun-and-verb semantic accuracy	4.91 (4.78–5.08)	5.74 (5.48–6.06)	5.07 (4.90–5.26)	6.00 (5.69–6.59)	5.72 (5.40–5.99)	5.92 (5.46–6.30)	<0.001
AlphaReadabilityChinese content-word semantic accuracy	5.35 (5.16–5.54)	6.12 (5.84–6.36)	5.49 (5.24–5.74)	6.29 (5.98–6.87)	6.11 (5.86–6.31)	6.31 (5.84–6.57)	<0.001
AlphaReadabilityChinese noun semantic richness	0.28 (0.27–0.30)	0.25 (0.24–0.27)	0.26 (0.24–0.26)	0.23 (0.20–0.25)	0.24 (0.23–0.27)	0.25 (0.23–0.28)	<0.001
AlphaReadabilityChinese noun semantic clarity	0.04 (0.03–0.05)	0.05 (0.04–0.06)	0.03 (0.02–0.04)	0.06 (0.04–0.07)	0.05 (0.04–0.07)	0.05 (0.04–0.06)	<0.001
AlphaReadabilityChinese noun semantic noise	24.04 (15.70–41.42)	18.13 (12.91–27.54)	22.61 (16.34–31.87)	17.56 (14.72–22.19)	17.00 (12.35–25.43)	13.94 (10.86–16.55)	<0.001
Chinese Readability Platform reading-difficulty score	15.86 (15.27–16.46)	14.09 (13.62–14.76)	16.77 (15.83–17.03)	13.16 (12.59–13.82)	13.88 (13.26–14.59)	13.94 (13.09–14.53)	<0.001
Actionability							
PEMAT-P Actionability score (%)	42.9 (28.6–42.9)	42.9 (28.6–42.9)	28.6 (14.3–42.9)	42.9 (28.6–42.9)	42.9 (28.6–42.9)	28.6 (14.3–42.9)	<0.001
Accuracy							
Clinical accuracy (Likert score, 1–5)	5.0 (5.0–5.0)	5.0 (5.0–5.0)	5.0 (4.0–5.0)	5.0 (4.0–5.0)	5.0 (5.0–5.0)	5.0 (4.0–5.0)	0.032
Safety							
Level 1 minor safety-related omissions, *n* (%)	5 (13.9%)	0 (0.0%)	5 (13.9%)	3 (8.3%)	1 (2.8%)	2 (5.6%)	0.084
Hallucination							
Responses with hallucination, *n* (%)	2 (5.6%)	0 (0.0%)	1 (2.8%)	0 (0.0%)	1 (2.8%)	0 (0.0%)	0.352

ARC, AlphaReadabilityChinese; CLDC, Chinese Language Difficulty Coefficient; EQIP-20, 20-item Ensuring Quality Information for Patients; GQS, Global Quality Score; PEMAT-P, Patient Education Materials Assessment Tool for Printable Materials; QAMAI, Quality Assessment of Medical Artificial Intelligence; Q1, first quartile; Q3, third quartile.

**Figure 2:**
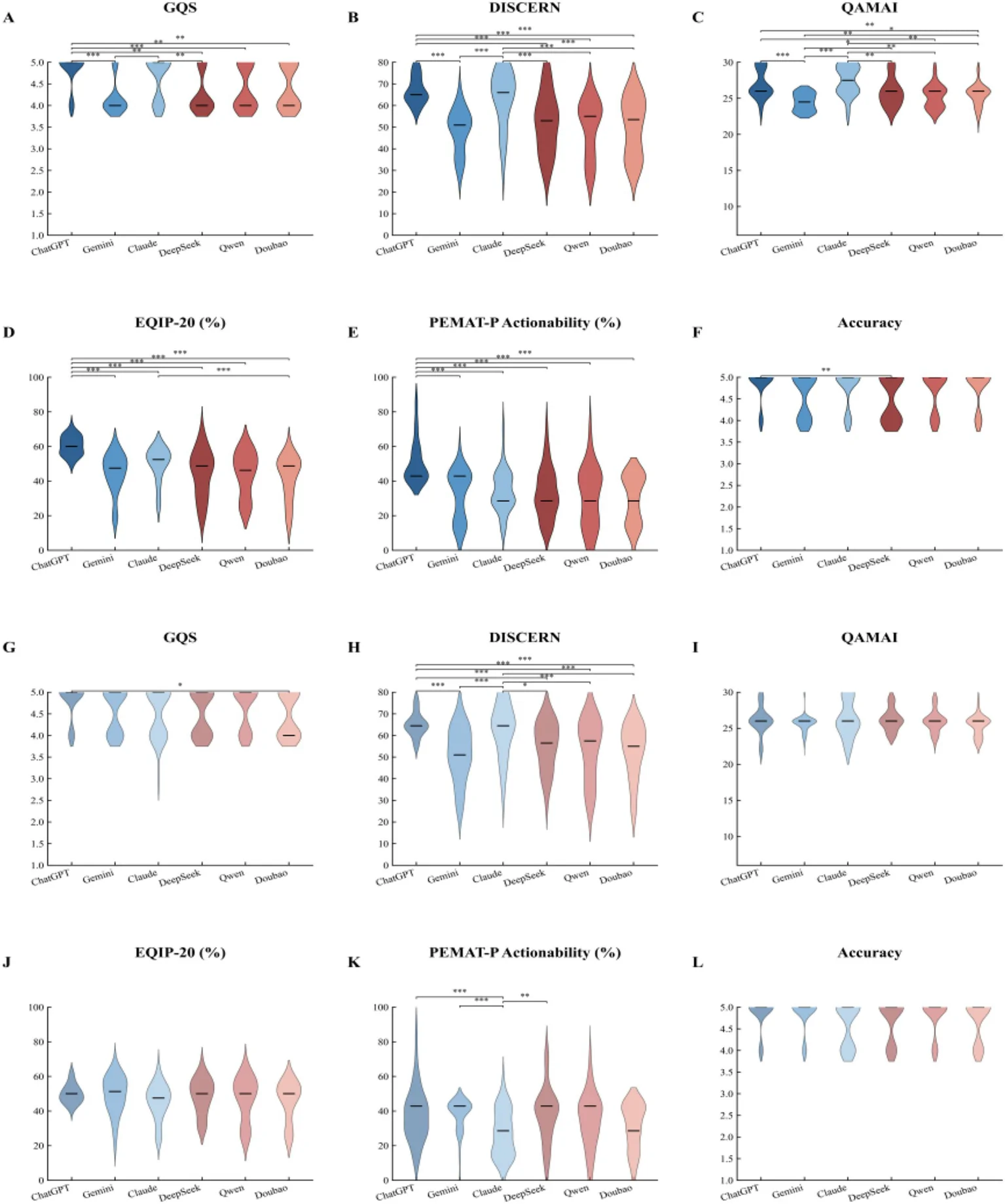
Distributions of response quality, actionability, and clinical accuracy by model and question language. **(A–F)** Responses to English questions: **(A)** Global Quality Score (GQS), **(B)** DISCERN total score, **(C)** Quality Analysis of Medical Artificial Intelligence (QAMAI) total score, **(D)** 20-item Ensuring Quality Information for Patients (EQIP-20) percentage score, **(E)** Patient Education Materials Assessment Tool for Printable Materials (PEMAT-P) actionability percentage score, and **(F)** clinical accuracy score. **(G–L)** Responses to Chinese questions: **(G)** GQS, **(H)** DISCERN total score, **(I)** QAMAI total score, **(J)** EQIP-20 percentage score, **(K)** PEMAT-P actionability percentage score, and **(L)** clinical accuracy score. *p < 0.05, **p < 0.01, and ***p < 0.001 indicate Holm-adjusted 2-sided *p*-values.

Item-level analyses of individual DISCERN and QAMAI sub-components were performed to examine whether global inter-model disparities in response quality were evenly distributed across all assessment sub-domains. Within English-language responses, inter-model differences were most marked for the treatment-related sub-items of DISCERN and the information-source dimension of QAMAI (Figures 3A,C). Among Chinese-language replies, DISCERN-score disparities were predominantly confined to treatment-related sub-items (Figure 3B). Importantly, despite the absence of a significant inter-model difference in the Chinese-language QAMAI total score (*p* = 0.395; Table 3), substantial inter-model heterogeneity was still evident in its information-source sub-domain (Figure 3D).

**Figure 3:**
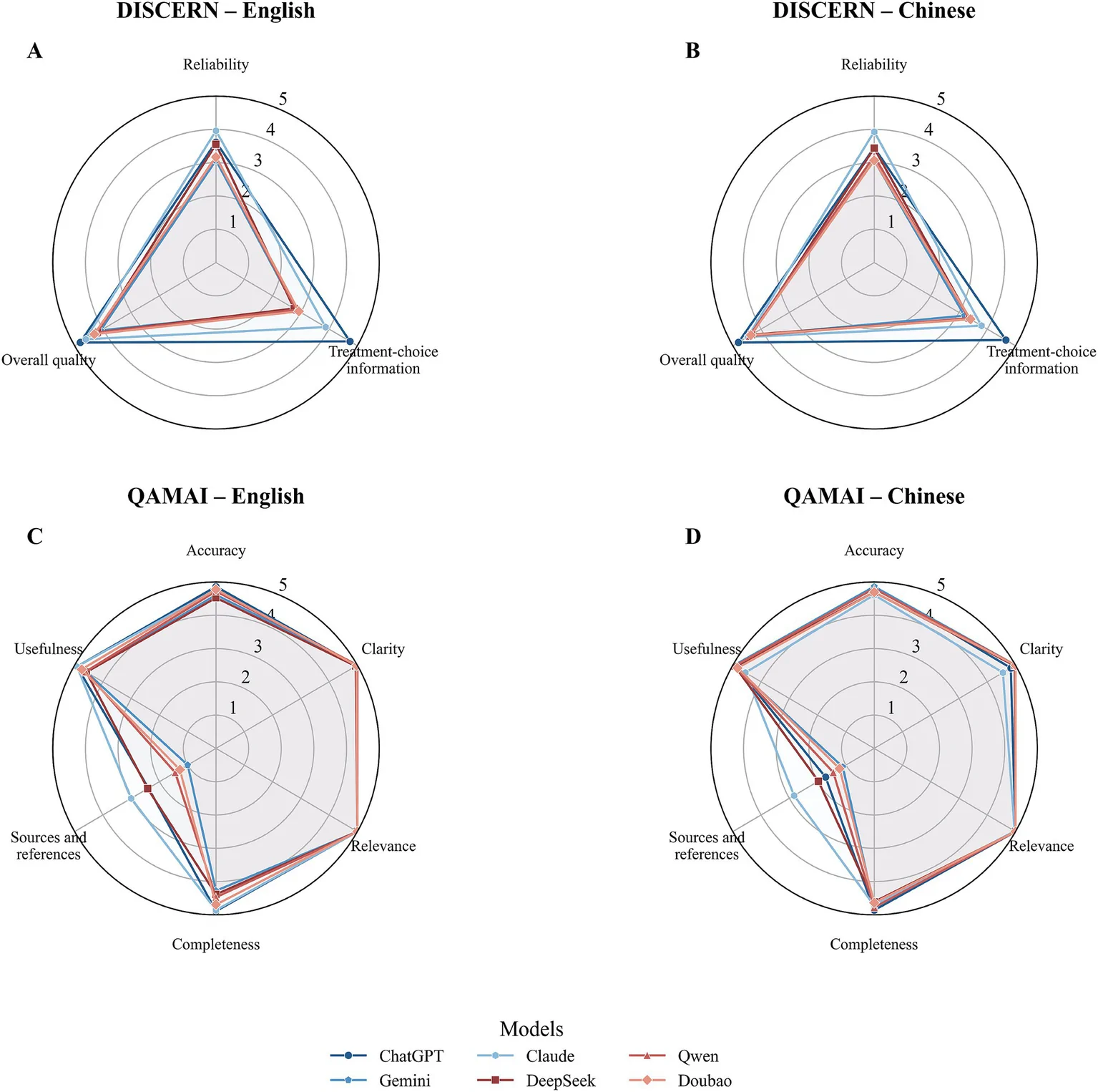
Model-level DISCERN and Quality Analysis of Medical Artificial Intelligence (QAMAI) profiles by question language. **(A)** DISCERN profile for responses to English questions. **(B)** DISCERN profile for responses to Chinese questions. **(C)** QAMAI item profile for responses to English questions. **(D)** QAMAI item profile for responses to Chinese questions.

### Analysis of cross-language response disparities

Cross-linguistic differences for GQS, DISCERN, QAMAI, EQIP-20, actionability, and clinical accuracy between English-oriented and Chinese-oriented LLMs are illustrated in Figure 4A. Across the six assessed endpoints, the English-Chinese difference in clinical-accuracy scores was closest to zero for both model-orientation subgroups, demonstrating that clinical accuracy displayed the smallest magnitude of cross-language variation (Figure 4A). In the exploratory orientation-by-language analysis, the observed cross-linguistic patterns differed between the two orientation groups for EQIP-20 and PEMAT-P actionability (Figure 4B). Given that each orientation group comprised only three independent models, these group-level patterns were interpreted descriptively and should not be considered evidence of a generalizable orientation effect.

**Figure 4:**
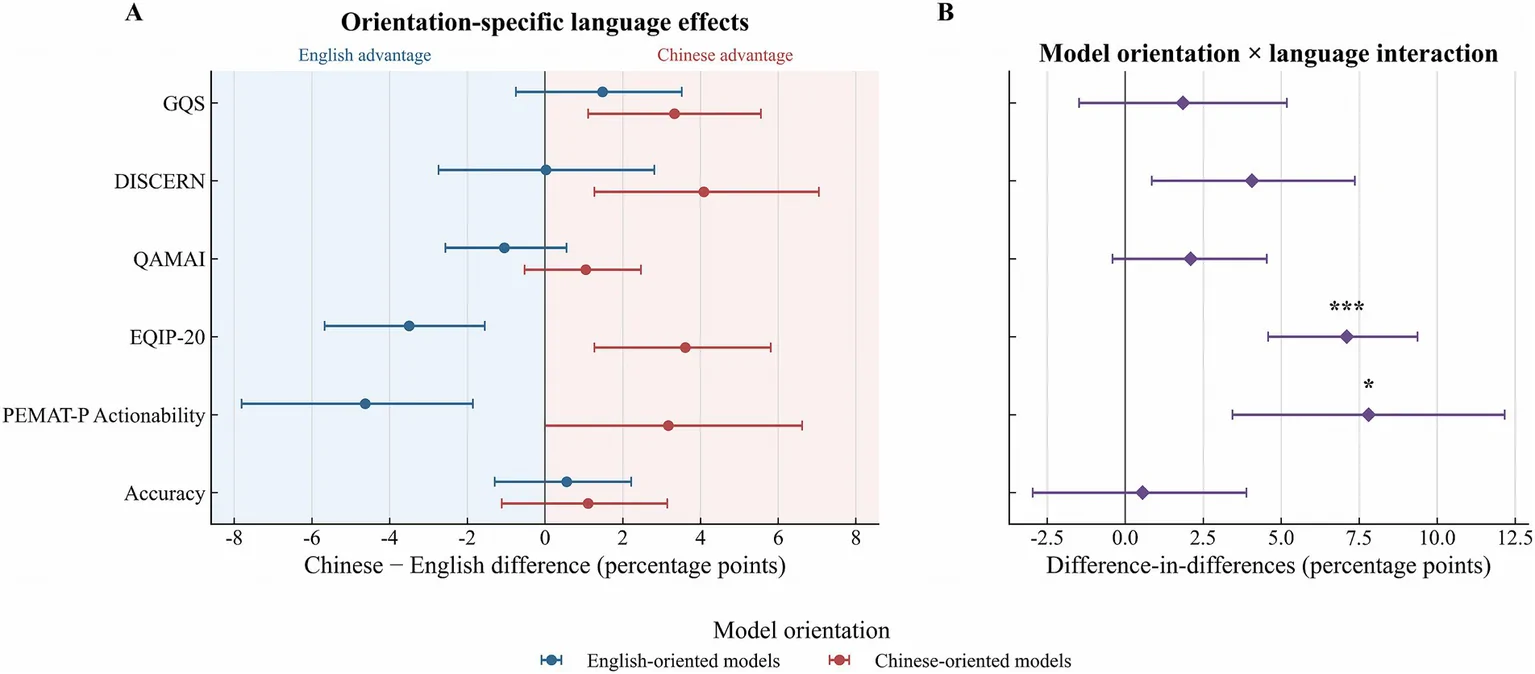
Cross-linguistic performance differences by model orientation. **(A)** Mean within-model differences between Chinese- and English-language responses in Global Quality Score (GQS), DISCERN total score, Quality Analysis of Medical Artificial Intelligence (QAMAI) total score, 20-item Ensuring Quality Information for Patients (EQIP-20) percentage score, Patient Education Materials Assessment Tool for Printable Materials (PEMAT-P) Actionability percentage score, and clinical accuracy score, stratified by model orientation. **(B)** Interaction contrasts calculated as the Chinese-minus-English difference among Chinese-oriented models minus the corresponding difference among English-oriented models. *p < 0.05, **p < 0.01, and ***p < 0.001.

Since aggregated group-level interaction statistics and pooled summary estimates may mask inter-model heterogeneity, we subsequently explored language-dependent changes in the six outcome metrics at the individual-model level when switching the response language from English to Chinese (Figure 5). For EQIP-20, ChatGPT-5.6-Sol experienced a pronounced decline after the language switch, while the remaining five models showed comparatively modest shifts. For PEMAT-P actionability, both ChatGPT-5.6-Sol and Claude-Fable-5 exhibited substantial reductions from English- to Chinese-language responses, whereas Gemini-3.1-Pro changed in the opposite direction. Cross-linguistic effect distributions were further stratified according to the six predefined thematic question domains (Supplementary material 1; Figure 6). Clinical accuracy remained largely stable across both languages within every domain. By contrast, the most prominent orientation-associated cross-language disparities in EQIP-20 and PEMAT-P actionability occurred in the Disease Foundations, Long-Term Planning, and Symptom Assessment domains (Figures 6D,E).

**Figure 5:**
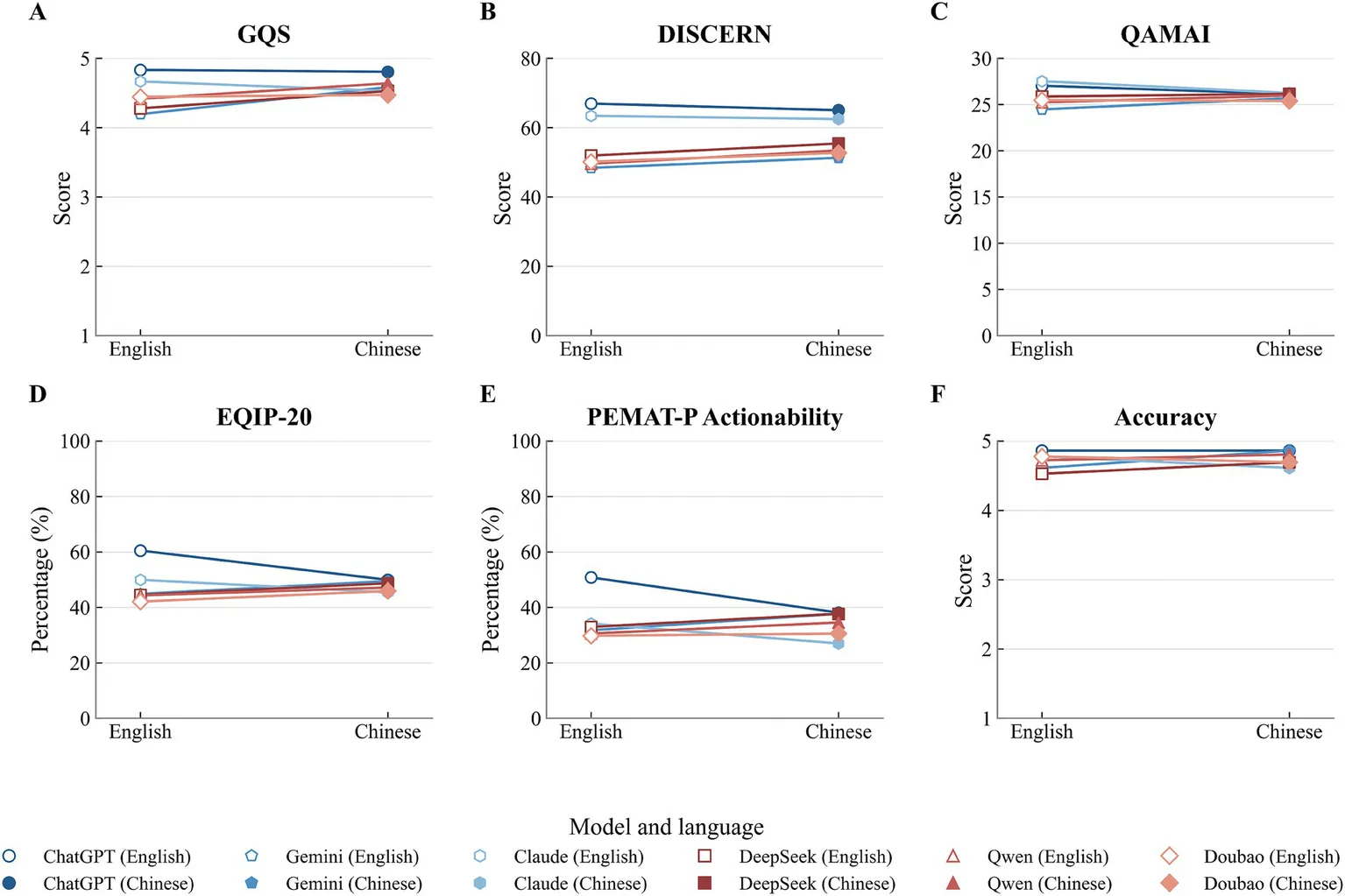
Model-level shifts in performance between English and Chinese responses.**(A)** Global Quality Score (GQS).**(B)** DISCERN total score.**(C)** Quality Analysis of Medical Artificial Intelligence (QAMAI) total score.**(D)** 20-item Ensuring Quality Information for Patients (EQIP-20) percentage score.**(E)** Patient Education Materials Assessment Tool for Printable Materials (PEMAT-P) Actionability percentage score.**(F)** Clinical accuracy score.

**Figure 6:**
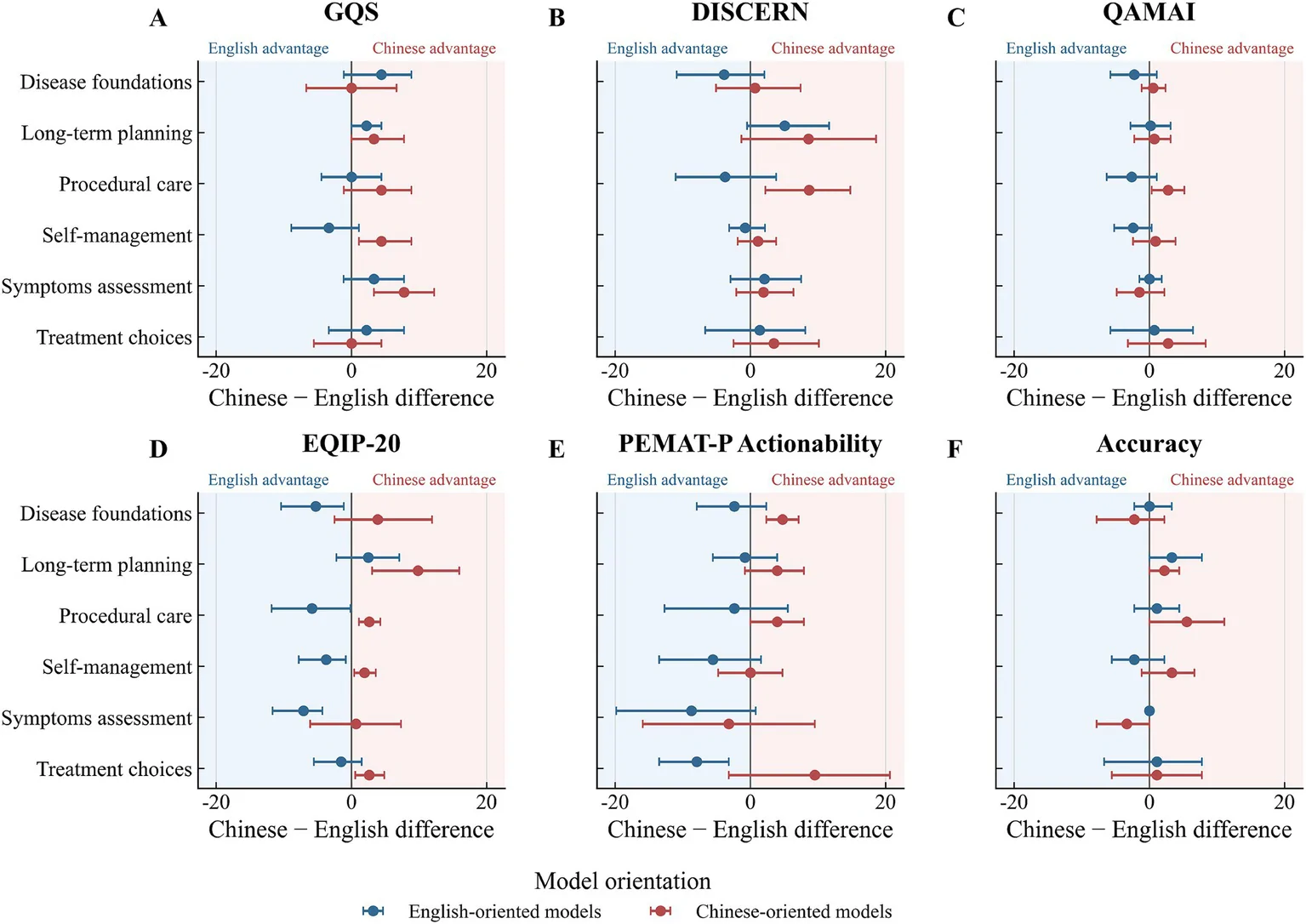
Domain-specific cross-linguistic differences in model performance. **(A)** Global Quality Score (GQS). **(B)** DISCERN total score. **(C)** Quality Analysis of Medical Artificial Intelligence (QAMAI) total score. **(D)** 20-item Ensuring Quality Information for Patients (EQIP-20) percentage score. **(E)** Patient Education Materials Assessment Tool for Printable Materials (PEMAT-P) actionability percentage score. **(F)** Clinical accuracy score. Within each of the 6 patient-question domains, points represent the mean Chinese-minus-English difference for English-oriented or Chinese-oriented models after each outcome was rescaled as a percentage of its maximum possible score. *p < 0.05, **p < 0.01, and ***p < 0.001.

### Clinical practicality and risk-related outcomes

As an exploratory visualization of the relationship between response actionability and the frequency of Level 1 minor safety-related omissions, a two-dimensional analysis was performed using PEMAT-P actionability scores and the proportion of responses classified as Level 1 for each model (Figure 7). The reference lines were included solely as descriptive visual aids and do not represent clinically validated thresholds. Under the English-language condition, ChatGPT-5.6-Sol was the only model situated in the high-actionability, low-minor-omission region. After switching to Chinese prompting, however, this model exhibited reduced actionability accompanied by an increased frequency of Level 1 minor omissions. In contrast, Claude-Fable-5 exhibited fewer Level 1 minor omissions when generating English-language than Chinese-language responses. The remaining models generally showed low frequencies of Level 1 minor omissions across both language settings, with only minor cross-linguistic variation, while maintaining relatively low actionability scores (Figure 7).

**Figure 7:**
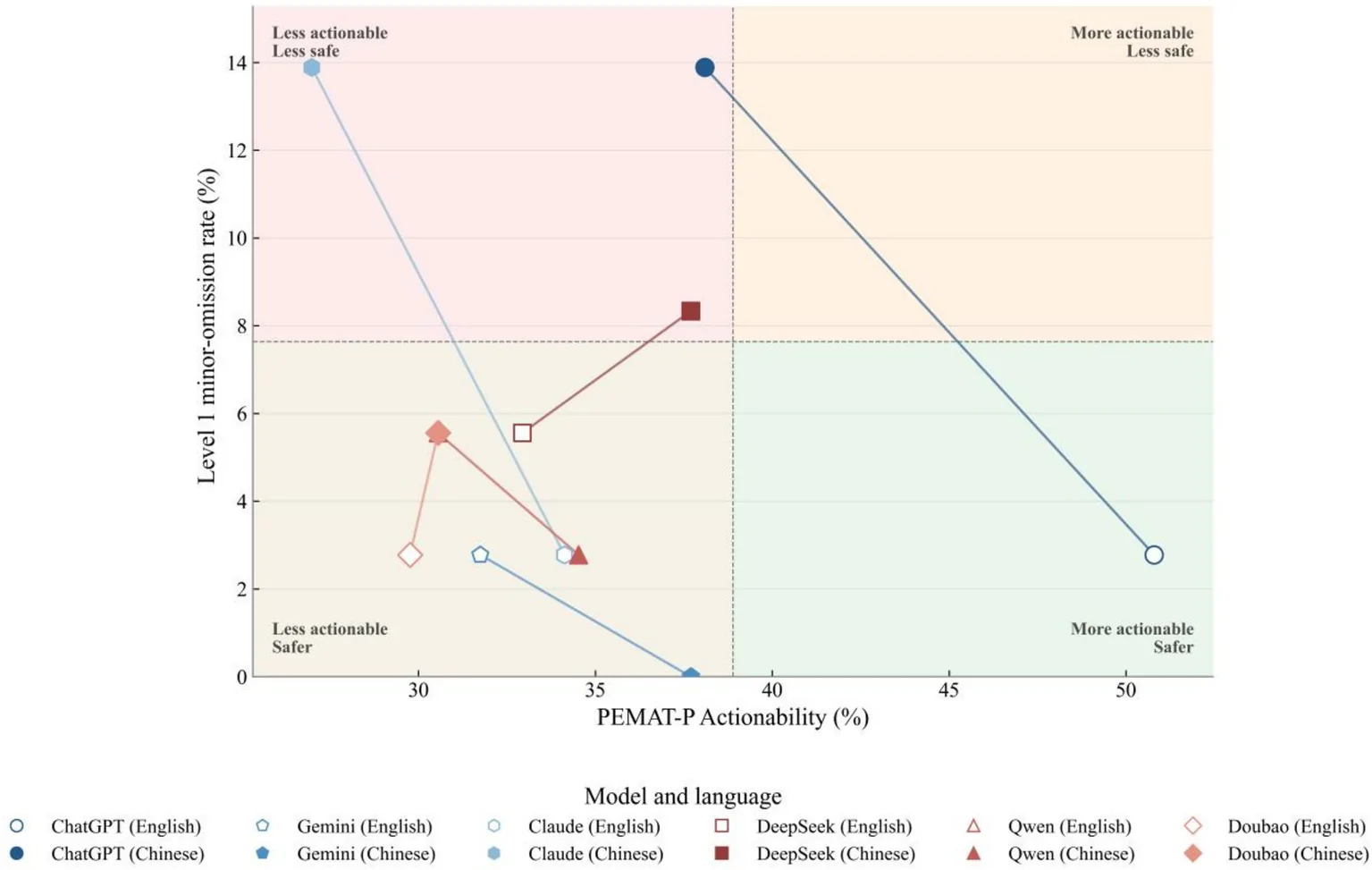
Actionability and Level 1 minor-omission rates by model and question language.

### Hallucination and safety-risk evaluation

All models were further evaluated for safety risk severity and hallucination incidence across English and Chinese response settings (Figure 8). No statistically significant inter-model differences were detected in the frequency of Level 1 minor safety-related omissions or hallucination rates within either language condition (all *p* > 0.05; Tables 2, 3). All observed nonzero safety events were classified as Level 1 minor safety-related omissions. No Level 2 or Level 3 events were identified in either language condition (Figure 8A). Descriptive comparisons revealed that ChatGPT-5.6-Sol yielded the highest observed hallucination rates under both English and Chinese conditions, whereas no hallucination events were observed for Gemini-3.1-Pro in either language condition (Figure 8B).

**Figure 8:**
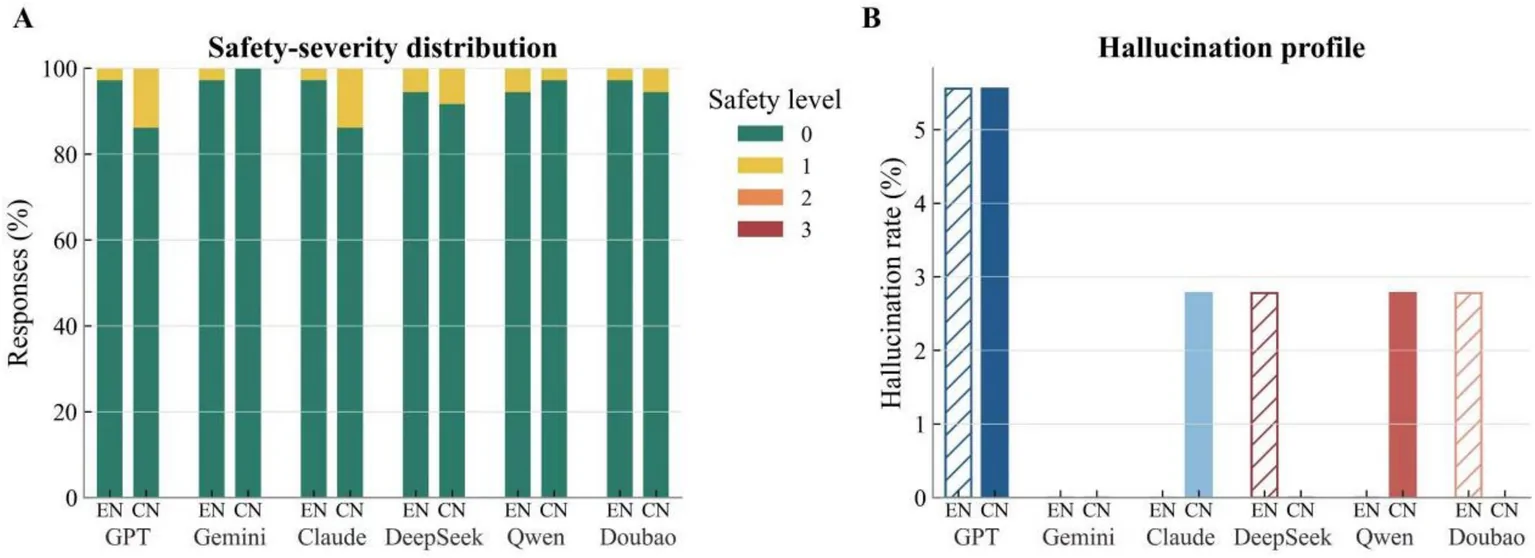
Safety and hallucination profiles by model and question language. **(A)** Percentage distribution of safety scores among the 36 responses in each model-language group. **(B)** Percentage of responses classified as containing hallucinations.

### Readability assessment

Significant inter-model differences in readability were detected among the six LLMs for both English- and Chinese-language outputs (all *p* < 0.001; Tables 2, 3). For English-language responses, ChatGPT-5.6-Sol exhibited the most favourable readability profile, achieving the highest median FRES score (38.25 [IQR, 33.73–41.70]) as well as the lowest median values for FKGL (11.92 [IQR, 11.07–12.85]), SMOG (13.91 [IQR, 13.17–14.70]), and GFI (15.22 [IQR, 14.15–16.43]) across all evaluated models (Table 2). By contrast, Doubao-Seed-2.1-Pro produced the least readable English-language outputs, with the lowest median FRES (12.05 [IQR, 0.73–20.36]) and the highest median FKGL (19.08 [IQR, 17.21–22.26]), SMOG (19.39 [IQR, 17.83–21.20]), and GFI (22.48 [IQR, 20.74–26.15]) scores (Table 2). Notably, none of the six models satisfied the predefined readability thresholds for English-language patient-education materials. Distributions of English-language readability metrics are illustrated in Figure 9.

**Figure 9:**
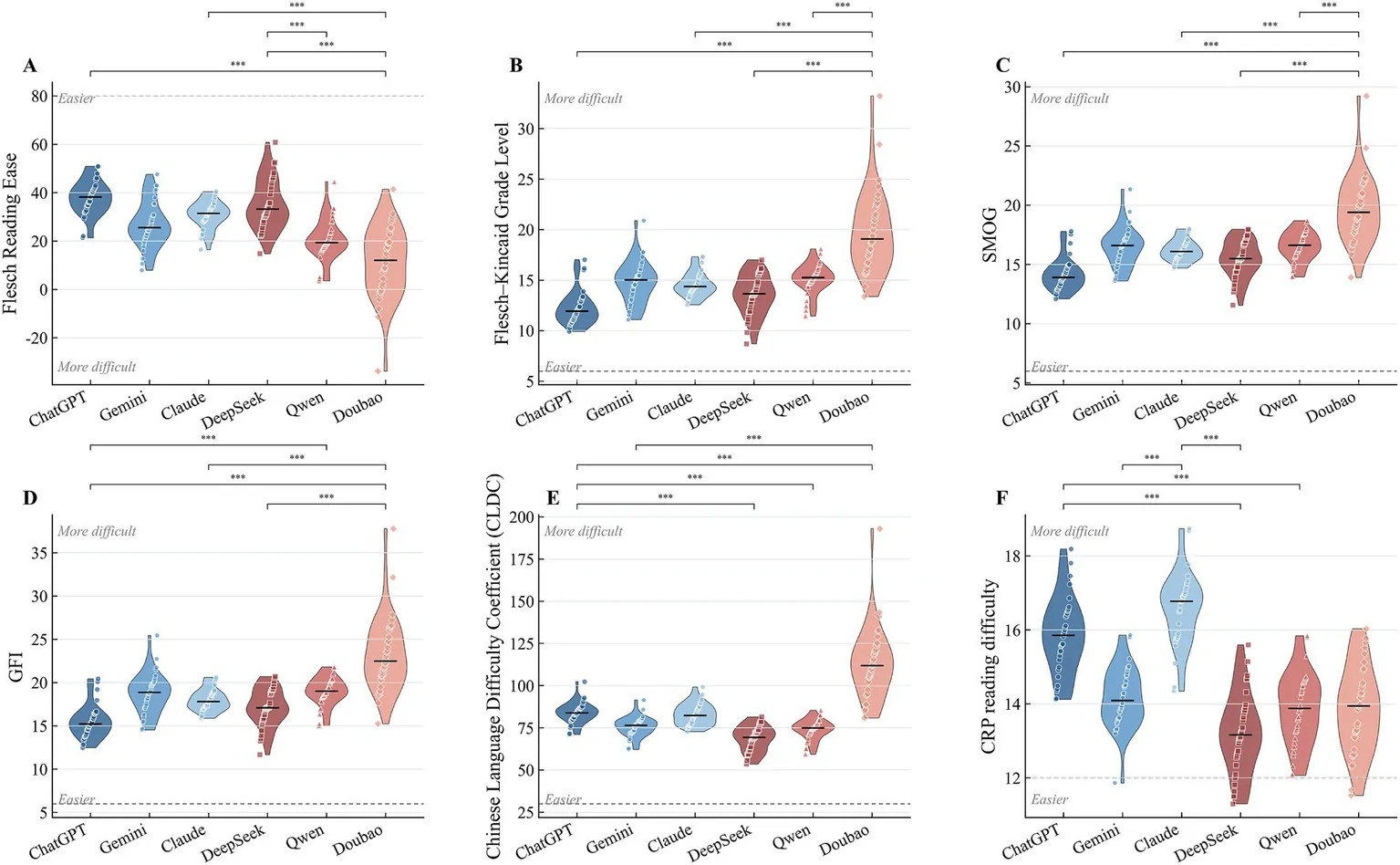
Distribution of language-specific readability measures across models. Responses to English questions: **(A)** Flesch Reading Ease Score (FRES), **(B)** Flesch–Kincaid Grade Level (FKGL), **(C)** Simple Measure of Gobbledygook (SMOG), and **(D)** Gunning Fog Index (GFI). Responses to Chinese questions: **(E)** Chinese Language Difficulty Coefficient (CLDC) and **(F)** Chinese Readability Platform (CRP) reading-difficulty score. *p < 0.05, **p < 0.01, and ***p < 0.001.

For Chinese-language responses, no model attained the prespecified Chinese readability benchmarks. DeepSeek-V4-Pro achieved the best overall readability performance, yielding the lowest median CLDC and CRP scores: 69.28 (IQR, 63.13–73.92) and 13.16 (IQR, 12.59–13.82), respectively (Table 3). Doubao-Seed-2.1-Pro again generated responses with the highest reading difficulty, with a median CLDC value of 111.86 (IQR, 101.20–121.83) (Table 3). Score distributions for Chinese-language readability outcomes across models are also presented in Figure 9.

Individual ARC sub-dimensions were analysed separately to further unpack the multidimensional features driving inter-model disparities in Chinese-language readability (Figure 10). Distinct linguistic patterns were identified across the six LLMs. DeepSeek-V4-Pro delivered comparatively favourable readability alongside high semantic accuracy. In contrast, the elevated reading difficulty observed in outputs from Doubao-Seed-2.1-Pro was characterized predominantly by increased lexical and syntactic complexity, rather than impaired semantic quality (Figure 10A). Furthermore, significant inter-model differences were observed across multiple surface-level linguistic features, especially indices reflecting lexical and syntactic richness (Figure 10).

**Figure 10:**
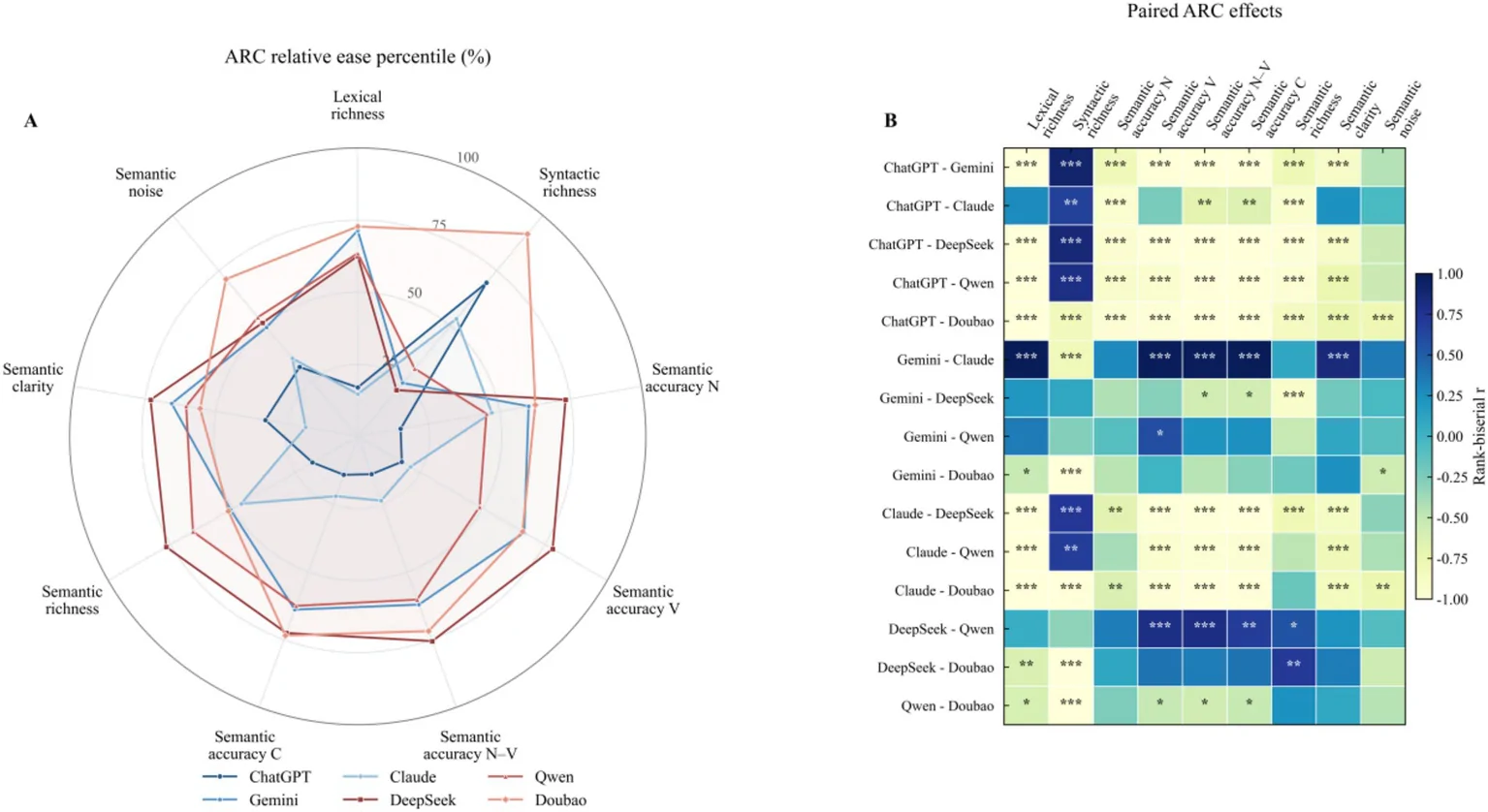
AlphaReadabilityChinese profiles and paired model contrasts for responses to Chinese questions. **(A)** Model-level median relative-ease percentiles across the 9 AlphaReadabilityChinese (ARC) dimensions. Scores were transformed to percentiles, with 0% indicating relatively greater difficulty and 100% indicating relatively greater ease. **(B)** Pairwise model effects for the same 9 ARC dimensions. Positive values favor the model listed first, whereas negative values favor the model listed second. *p < 0.05, **p < 0.01, and ***p < 0.001.

### Correlation analysis of evaluation indicators

Spearman correlation analyses were performed to explore the pairwise associations between information quality metrics and readability features across bilingual settings (Figure 11). Although trend fitting suggested potential covariation between response quality and readability, no statistically significant monotonic correlations were identified for either English-oriented or Chinese-oriented models in either language mode (all *p* > 0.05; Figure 11). For English-language outputs, ChatGPT-5.6-Sol and Claude-Fable-5 achieved superior median scores in both DISCERN and FRES, demonstrating a descriptive concordance between high information quality and favorable readability (Figure 11C). In contrast, when generating Chinese-language responses, these two models maintained the highest DISCERN scores but exhibited elevated CR*p* values, which correspond to greater reading difficulty (Figure 11D). This distinct bilingual correlation pattern indicates a notable cross-linguistic divergence between information quality and readability performance.

**Figure 11:**
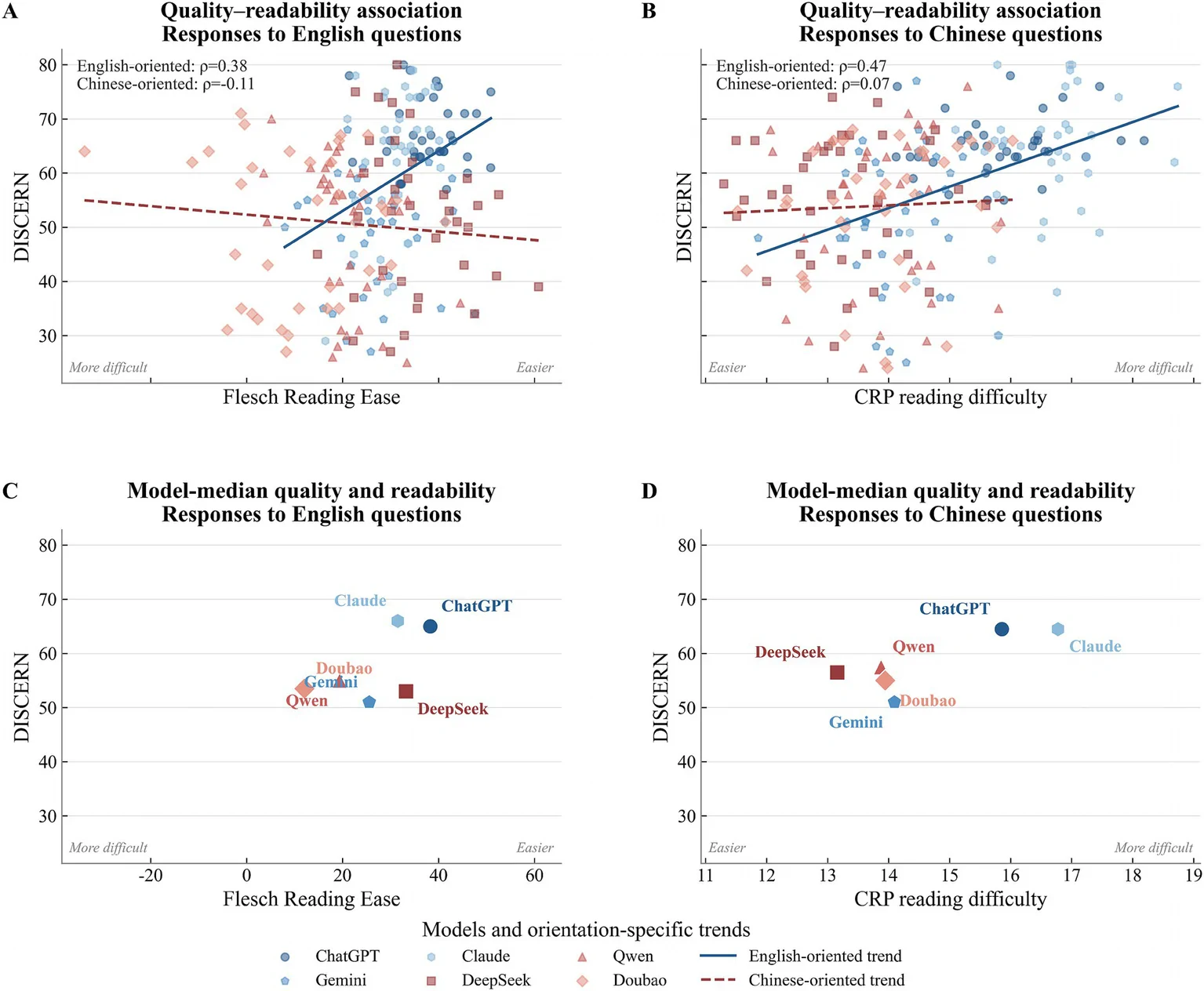
Associations between response quality and language-specific readability. **(A)** Response-level association between DISCERN score and Flesch Reading Ease Score (FRES) for answers to English questions. **(B)** Response-level association between DISCERN score and Chinese Readability Platform (CRP) reading-difficulty score for answers to Chinese questions. **(C)** Model-level median DISCERN score and median FRES for responses to English questions. **(D)** Model-level median DISCERN score and median CRP reading-difficulty score for responses to Chinese questions.

The correlation structure among the evaluated constructs, including information quality, actionability, clinical accuracy, safety, hallucination occurrence, and readability, was further explored (Figure 12). In both English- and Chinese-language responses, GQS, DISCERN, QAMAI, and EQIP-20 demonstrated significant positive correlations with each other (*p* < 0.05; Figures 12A,B), except for the correlation between QAMAI and EQIP-20 under the Chinese-language condition, which did not reach statistical significance. These findings suggested good convergence among the four information quality assessment instruments. No significant correlations were observed between actionability and either clinical accuracy or safety-risk severity under either language condition (*p* > 0.05; Figures 12A,B).

**Figure 12:**
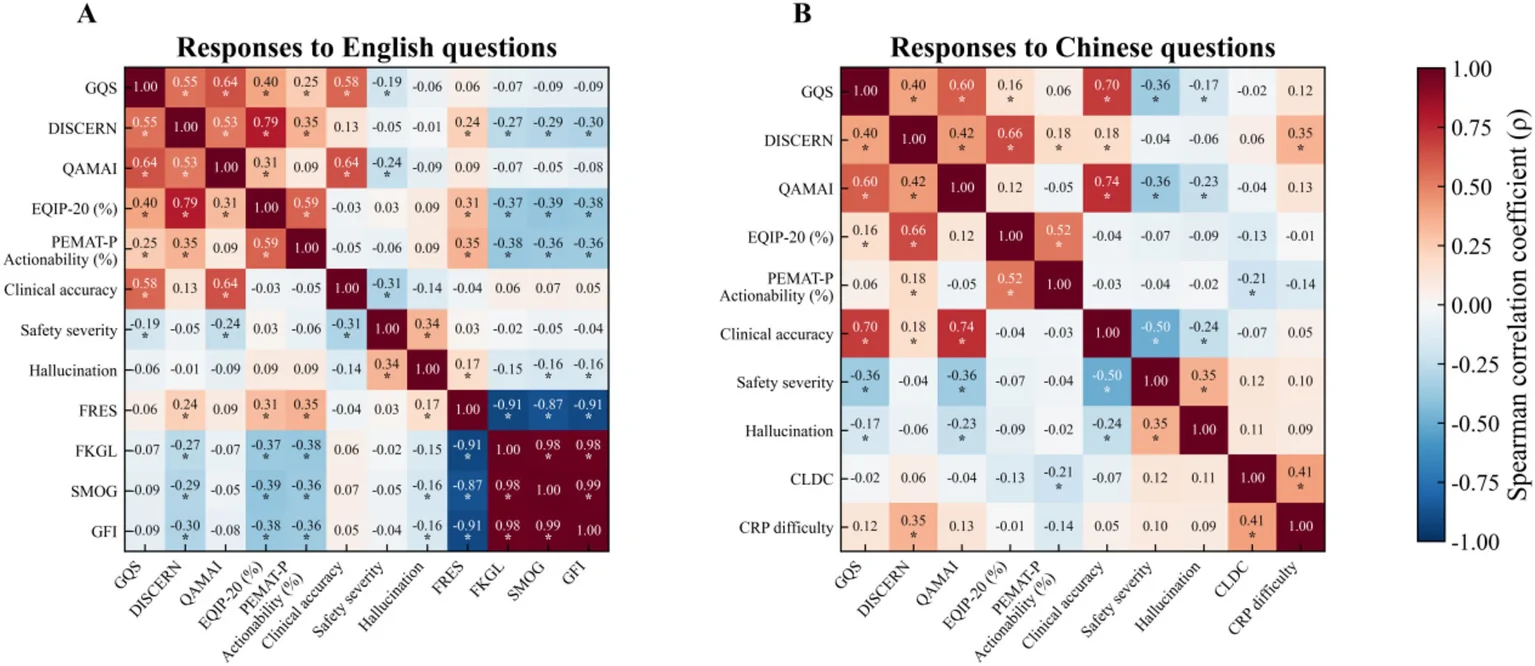
Correlation structure of response-evaluation measures by question language. **(A)** Spearman correlation matrix for responses to English questions, including Global Quality Score (GQS), DISCERN total score, Quality Analysis of Medical Artificial Intelligence (QAMAI) total score, 20-item Ensuring Quality Information for Patients (EQIP-20) percentage score, Patient Education Materials Assessment Tool for Printable Materials (PEMAT-P) actionability percentage score, clinical accuracy, safety severity, hallucination, Flesch Reading Ease Score (FRES), Flesch–Kincaid Grade Level (FKGL), Simple Measure of Gobbledygook (SMOG), and Gunning Fog Index (GFI). **(B)** Spearman correlation matrix for responses to Chinese questions, including GQS, DISCERN total score, QAMAI total score, EQIP-20 percentage score, PEMAT-P actionability percentage score, clinical accuracy, safety severity, hallucination, Chinese Language Difficulty Coefficient (CLDC), and Chinese Readability Platform (CRP) reading-difficulty score. *p < 0.05.

Under the English-language condition, information quality measures showed weak correlations with safety-risk severity but were not significantly associated with hallucination occurrence (Figure 12A). Under the Chinese-language condition, information quality measures demonstrated weak correlations with both safety-risk severity and hallucination occurrence (Figure 12B). Furthermore, readability was not significantly correlated with either clinical accuracy or safety-risk severity in either English- or Chinese-language responses (*p* > 0.05; Figures 12A,B).

## Discussion

### Interpretation of main findings

In this study, we simulated patient-oriented health consultations concerning ONFH using semantically matched English-Chinese question pairs and comprehensively evaluated health-related outputs generated by six state-of-the-art large language models across multiple clinically relevant dimensions, including information quality, readability, actionability, clinical accuracy, and safety-risk profiles. Under the English-language condition, significant inter-model differences were detected for all four information-quality metrics, as well as for actionability and clinical accuracy (Tables 2, 3). After switching to Chinese prompting, inter-model disparities remained significant across most endpoints, with the notable exception of the total QAMAI score. Item-level analyses further revealed that inter-model differences in DISCERN scores were predominantly concentrated on treatment-related content in both language settings. These items cover clinically critical components of ONFH management, including stage-stratified hip-preserving strategies and surgical indications for total hip arthroplasty, and thus constitute an essential facet of patient-targeted ONFH health information. From a clinical perspective, accurate and comprehensive communication of treatment-related information is particularly important in ONFH because management decisions depend substantially on disease stage, structural progression, and treatment eligibility. Incomplete or unclear communication of these considerations may limit patients’ understanding of the distinction between joint-preserving treatment for earlier-stage disease and arthroplasty-based management for advanced structural deterioration. Notably, although no significant inter-model difference was found for the Chinese-language QAMAI total score, considerable residual variation persisted within the information-source sub-dimension. This observation highlights a key limitation of relying solely on aggregated summary scores for LLM evaluation, since composite metrics may mask clinically meaningful deficits at the individual-subscale level.

### Cross-linguistic performance differences

A second notable finding was the substantial heterogeneity in cross-linguistic performance changes across the evaluated LLMs. The exploratory orientation-by-language analysis suggested different group-level patterns for EQIP-20 and PEMAT-P actionability; however, these observations should not be interpreted as evidence of a generalizable model-orientation effect because only three independent models were represented within each orientation group. Accordingly, greater interpretative emphasis should be placed on cross-linguistic changes observed within individual models. Uneven linguistic representation within training corpora may provide one possible explanation for such model-specific variation, as models may perform differently across languages depending on the linguistic composition of their training data and language-specific optimization (55). Yao et al. similarly reported differences in Chinese-language performance between Chinese-oriented LLMs and ChatGPT, highlighting the potential influence of localized training resources (56). Collectively, these observations highlight the potential for cross-linguistic performance variation among the LLMs evaluated in the present study and support the need for broader multilingual evaluations involving a larger and more diverse sample of models (57). Importantly, clinical accuracy showed relatively small cross-linguistic variation compared with the other evaluated dimensions, suggesting relative stability of factual clinical content across languages within the evaluated models. This pattern may reflect the standardized and evidence-based nature of core biomedical knowledge. Nevertheless, because the orientation groups comprised only three models each and question-level responses represented repeated observations within models rather than independent model-level replications, the orientation-level findings should be regarded as descriptive and exploratory and should not be extrapolated beyond the models evaluated in this study.

Given these considerations, greater emphasis was placed on individual-model patterns of cross-linguistic performance change when interpreting language-related differences. Model-stratified analyses further uncovered substantial inter-model heterogeneity in cross-linguistic performance changes across assessment domains. ChatGPT-5.6-Sol showed a pronounced reduction in EQIP-20 scores when responses were generated in Chinese rather than English. Similarly, PEMAT-P actionability declined for both ChatGPT-5.6-Sol and Claude-Fable-5, whereas Gemini-3.1-Pro displayed performance change in the opposite direction. These divergent individual-model patterns indicate that the exploratory orientation-group differences in EQIP-20 and PEMAT-P actionability were not uniformly expressed across models and may have been influenced by marked cross-linguistic shifts in a subset of individual LLMs. Domain-stratified subgroup analyses demonstrated that cross-linguistic effects were not evenly distributed across the six predefined clinical topic domains. Clinical accuracy remained comparatively stable across all thematic categories, further suggesting relative cross-linguistic stability in factual knowledge outputs. In contrast, the exploratory group-level cross-linguistic differences in EQIP-20 and PEMAT-P actionability were most apparent within the Disease Foundations, Long-Term Planning, and Symptom Assessment domains. These thematic areas frequently demand elaborate explanations of complex medical terminology, multi-step actionable guidance, and nuanced symptom descriptions, imposing higher requirements on linguistic organisation and expressive precision and potentially rendering these outputs more vulnerable to cross-linguistic performance decay.

### Clinical implications

The exploratory two-dimensional analysis of actionability and Level 1 minor safety-related omission rates further illustrated heterogeneous cross-linguistic patterns across individual models. Under the English-language condition, ChatGPT-5.6-Sol exhibited relatively high actionability and a low frequency of Level 1 minor safety-related omissions; upon switching to Chinese, however, its actionability decreased while the frequency of such omissions increased. Together with the pronounced cross-linguistic declines observed in EQIP-20 and PEMAT-P actionability, these findings indicate that language-related performance changes in ChatGPT-5.6-Sol occurred across multiple evaluation dimensions. Conversely, Claude-Fable-5 exhibited a lower frequency of Level 1 minor safety-related omissions when generating English-language than Chinese-language responses, further illustrating the heterogeneity of cross-linguistic response patterns across individual models. Such heterogeneity may reflect differences in pretraining datasets, architectural design, and language-specific safety-alignment procedures (58). The remaining evaluated models generally exhibited low frequencies of Level 1 minor safety-related omissions in both languages but relatively low actionability scores, highlighting the challenge of generating patient-facing medical information that is both practically actionable and sufficiently comprehensive to minimize safety-related omissions. Importantly, all observed nonzero safety events were Level 1 minor omissions, and no Level 2 or Level 3 unsafe responses were identified. Accordingly, the cross-linguistic variation observed in Figure 7 reflects differences in the frequency of minor safety-related omissions rather than differences in clinically consequential unsafe responses.

Although no statistically significant inter-model differences in safety outcomes were detected, the low number of events limits statistical power and precludes interpreting these findings as evidence of equivalent safety performance across models. Nevertheless, medical hallucination remains an important consideration in the use of LLMs for patient education (59). Prior investigations have reported that approximately one-third of LLM-generated disease-specific health recommendations deviate from authoritative clinical practice guidelines (60). In the present study, ChatGPT-5.6-Sol descriptively exhibited the highest hallucination rates under both language conditions, while hallucination events were exceedingly rare for Gemini-3.1-Pro. Although these descriptive trends may reflect underlying differences in model architecture, training-data composition, retrieval-augmented workflows, or post-training alignment, the absence of statistically significant inter-model differences precludes definitive conclusions regarding comparative hallucination-resilience among the tested systems.

Readability constitutes another critical determinant of the accessibility and suitability of patient-targeted health materials (61). Consensus patient-education guidelines commonly recommend that printed resources should be written at or below a sixth-grade reading level (62, 63). None of the six evaluated LLMs satisfied our prespecified readability benchmarks for English-language patient education. ChatGPT-5.6-Sol delivered the most readable English outputs, yet its median Flesch–Kincaid Grade Level corresponded roughly to a 12th-grade reading requirement. This finding aligns broadly with previous work reporting that unmodified outputs from ChatGPT-family models typically demand approximately a 12th-grade reading level (16, 64). By comparison, Doubao-Seed-2.1-Pro produced the least readable English-language responses, with a median FKGL score near grade 19, far exceeding standard patient-education readability recommendations. Such inter-model readability disparities may originate from differences in pretraining-corpus linguistic features and instruction-tuning protocols (65, 66). Models primarily trained on Chinese-language corpora may, when tasked with English-language generation, produce syntactically complex sentences and infrequent vocabulary items, thereby increasing text-reading difficulty (67).

A similar accessibility gap was observed for Chinese-language outputs: none of the six LLMs achieved the predefined Chinese-language readability thresholds. DeepSeek-V4-Pro yielded the best overall Chinese readability performance, whereas Doubao-Seed-2.1-Pro again generated the most text-difficult responses, demonstrating that its tendency to produce linguistically complex text was not limited to English-language generation. Multi-dimensional sub-dimension analysis using the AlphaReadabilityChinese (ARC) toolkit further revealed that the elevated reading difficulty of Doubao-Seed-2.1-Pro-generated Chinese-language content was driven predominantly by increased lexical richness and syntactic complexity, rather than impaired semantic clarity or excessive semantic noise. Cross-model comparisons similarly indicated that higher reading difficulty was associated with surface-level linguistic complexity instead of deeper-level semantic ambiguity or redundant information. These results suggest that cross-LLM differences in Chinese-language readability arise, at least partially, from variation in linguistic expression rather than substantive differences in underlying semantic content.

The four information-quality assessment tools were significantly positively correlated with one another in both language conditions, with the single exception of the QAMAI-EQIP-20 correlation under Chinese-language prompting. This convergent pattern demonstrates substantial agreement across these complementary instruments when appraising overall information quality, notwithstanding their divergent primary evaluation domains (44, 68). The observed inter-instrument convergence also supports the methodological benefit of deploying multiple, mutually complementary assessment tools when benchmarking medical-information outputs from LLMs (69–70). Critically, actionability showed no significant correlation with either clinical accuracy or safety-risk severity. Consequently, highly actionable, easy-to-follow LLM-derived recommendations do not inherently guarantee superior clinical accuracy or improved safety. Comparable dissociation phenomena have been documented in LLM-assisted simplification of hospital discharge summaries, in which improved readability did not eliminate residual deficits in factual accuracy and safety (71). This important dissociation carries direct clinical implications for patient-facing deployment: clearly worded, actionable advice may increase patient adherence, which could amplify potential harm if the underlying clinical content is inaccurate or unsafe.

Likewise, readability was not statistically associated with clinical accuracy or safety-risk severity in either English- or Chinese-language responses. Consistent with our findings, Alamleh et al. evaluated patient-education resources for Ménière disease and reported no significant association between text readability and the overall quality or reliability of health information (72). Moreover, evidence from a randomised controlled trial indicated that lowering text-reading difficulty alone does not reliably improve patients’ factual medical knowledge acquisition (73). Taken collectively, these observations confirm that linguistic accessibility and factual-clinical quality represent related yet distinct dimensions of patient-centred health communication. Optimising readability can reduce health-literacy burdens for end-user patients, yet readability improvements alone cannot guarantee factual correctness or eliminate unsafe clinical guidance.

### Study limitations

Several methodological limitations must be acknowledged when interpreting the present findings. First, the custom-built question bank was restricted exclusively to patient consultations for ONFH, a single orthopaedic disease. Accordingly, results may not generalise to other disease categories, alternative clinical contexts, different language pairs, or distinct patient populations. Although the question bank underwent specialist review for clinical relevance, patients were not directly involved in question selection or formal content validation; therefore, its representativeness of the actual information needs of patients with ONFH remains to be established. Second, each model–language–question combination was represented by a single response generated under a standardized single-shot prompting protocol; therefore, the findings should be interpreted as a snapshot of model performance under the study conditions and do not capture within-model generation-to-generation variability. Third, this investigation performed objective text-level evaluation of LLM outputs and did not directly measure real-patient comprehension, health-decision quality, self-management behaviours, or downstream clinical outcomes. Fourth, only three LLMs were included in each predefined model-orientation group, limiting independent model-level replication for orientation-group comparisons. Because question-level responses were nested within individual models rather than representing independent replications of model orientation, the orientation-by-language findings should be considered exploratory and interpreted cautiously. Finally, although clinical accuracy was assessed against question-specific reference standards comprising predefined key clinical elements, the present study focused on standardized quantitative comparisons and did not prospectively code specific categories of question-level omissions or inaccuracies across individual model responses. In addition, the involvement of the same clinical experts in developing the question-specific reference standards and evaluating LLM-generated responses may have introduced potential assessor-related bias, despite the use of predefined clinical criteria and independent scoring procedures. Accordingly, the aggregate accuracy scores should not be interpreted as identifying particular ONFH-specific error types.

### Future research

Future studies should extend the present evaluation framework to a broader range of orthopaedic conditions, clinical scenarios, and language pairs, with direct patient involvement in question-bank development and validation to better reflect real-world patient information needs. Repeated independent response generation across different model versions and time points is warranted to assess response reproducibility and temporal stability. Prospective studies involving patients should also investigate whether observed differences in information quality, readability, actionability, and clinical accuracy translate into differences in patient comprehension, decision-making, self-management behaviours, and clinical outcomes. Furthermore, evaluations involving larger and more diverse samples of LLMs are needed to more robustly investigate potential associations between model language orientation and cross-linguistic performance. Finally, predefined item-level error taxonomies and qualitative analyses could help characterize clinically relevant omissions and inaccuracies. Language-specific readability optimization strategies should also be evaluated to improve the accessibility of LLM-generated patient education materials.

## Conclusion

In conclusion, state-of-the-art LLMs demonstrated substantial heterogeneity in the quality, actionability, readability, and cross-linguistic consistency of patient-oriented information regarding ONFH, despite generally favourable clinical accuracy. Observed safety-related events were limited to Level 1 minor omissions, with no Level 2 or Level 3 events identified; however, these findings do not establish equivalent safety performance across models. Cross-linguistic performance differences were most pronounced for EQIP-20 and PEMAT-P actionability, with the magnitude and direction of these changes varying across individual models and clinical domains, whereas clinical accuracy remained comparatively stable across languages. Importantly, neither readability nor actionability reliably reflected clinical accuracy or safety, underscoring the need for multidimensional rather than single-metric evaluation of LLM-generated patient education materials. Although current LLMs show considerable potential as supplementary tools for ONFH patient education, their outputs remain insufficiently readable for general patient populations and should not be assumed to be clinically reliable solely on the basis of clarity or actionability. Future model development should prioritize multilingual consistency, patient-appropriate readability, evidence transparency, and clinically validated safety before widespread implementation in patient-facing orthopaedic care.

## Data Availability

The raw data supporting the conclusions of this article will be made available by the authors, without undue reservation.
