# Supplementary material for: Cross-linguistic differences in responses to patient queries on osteonecrosis of the femoral head between English and Chinese-oriented large language models: a semantically matched bilingual cross-sectional study
Source: Front Public Health. 2026 Oct 28;14:1985180. doi: 10.3389/fpubh.2026.1985180 (PMC13752189; doi:10.3389/fpubh.2026.1985180)
Supplement: Supplementary file 1 [file Table_1.docx]

**Supplementary Material 1. Bilingual Patient Question Bank for Osteonecrosis of the Femoral Head**

**Supplementary Table S1a. English Patient Question Bank for Osteonecrosis of the Femoral Head**

| **No.** | **Patient question** |
| --- | --- |
| **Disease foundations** | |
| 1 | What is osteonecrosis of the femoral head, and how can it damage my hip? |
| 2 | Which factors can increase my risk of osteonecrosis of the femoral head? |
| 3 | Can osteonecrosis of the femoral head affect both hips? |
| 4 | Can osteonecrosis of the femoral head be present before I feel hip pain? |
| 5 | How do my medical history and medicines affect the evaluation of osteonecrosis of the femoral head? |
| 6 | Why is the stage of osteonecrosis of the femoral head important for treatment planning? |
| **Symptoms assessment** | |
| 7 | What symptoms are common in osteonecrosis of the femoral head? |
| 8 | When should pain from osteonecrosis of the femoral head be assessed urgently? |
| 9 | Which tests are used to diagnose osteonecrosis of the femoral head? |
| 10 | Why might an X-ray be normal in early osteonecrosis of the femoral head? |
| 11 | What does magnetic resonance imaging show in osteonecrosis of the femoral head? |
| 12 | How will my clinician check whether osteonecrosis of the femoral head has caused collapse or arthritis? |
| **Treatment choices** | |
| 13 | What treatment options are available for osteonecrosis of the femoral head? |
| 14 | How does the stage of osteonecrosis of the femoral head affect treatment choices? |
| 15 | What is core decompression for osteonecrosis of the femoral head? |
| 16 | When might total hip replacement be considered for osteonecrosis of the femoral head? |
| 17 | What should I know about medicines for pain from osteonecrosis of the femoral head? |
| 18 | How can I compare the benefits and risks of treatment options for osteonecrosis of the femoral head? |
| **Self-management** | |
| 19 | How should I adjust weight-bearing activities with osteonecrosis of the femoral head? |
| 20 | Which exercises are usually appropriate for osteonecrosis of the femoral head? |
| 21 | Can a cane, crutches, or another walking aid help with osteonecrosis of the femoral head? |
| 22 | Should I stop corticosteroid medicine if I have osteonecrosis of the femoral head? |
| 23 | How should alcohol use be discussed when I have osteonecrosis of the femoral head? |
| 24 | How can I manage daily activities when osteonecrosis of the femoral head limits my hip movement? |
| **Procedural care** | |
| 25 | What follow-up is usually needed after core decompression for osteonecrosis of the femoral head? |
| 26 | What recovery milestones should I discuss after total hip replacement for osteonecrosis of the femoral head? |
| 27 | What complications should I discuss before surgery for osteonecrosis of the femoral head? |
| 28 | When can I return to work, driving, or sport after treatment for osteonecrosis of the femoral head? |
| 29 | Which symptoms after surgery for osteonecrosis of the femoral head need urgent medical attention? |
| 30 | How will osteonecrosis of the femoral head be monitored if I do not have surgery now? |
| **Long-term planning** | |
| 31 | Can osteonecrosis of the femoral head heal or reverse without treatment? |
| 32 | What factors affect the chance that osteonecrosis of the femoral head will progress or collapse? |
| 33 | How can osteonecrosis of the femoral head affect my mood, sleep, or daily life? |
| 34 | Why is shared decision-making important for osteonecrosis of the femoral head? |
| 35 | What information should I bring to an appointment about osteonecrosis of the femoral head? |
| 36 | How can I make a long-term plan for osteonecrosis of the femoral head with my care team? |

**Supplementary Table S1b. Chinese Patient Question Bank for Osteonecrosis of the Femoral Head**

| **序号** | **患者问题** |
| --- | --- |
| **病情基础** | |
| 1 | 什么是股骨头坏死，它会怎样损害我的髋关节？ |
| 2 | 哪些因素会增加我发生股骨头坏死的风险？ |
| 3 | 股骨头坏死会同时累及两侧髋关节吗？ |
| 4 | 在我感觉到髋痛之前，股骨头坏死会不会已经存在？ |
| 5 | 我的病史和正在使用的药物会怎样影响股骨头坏死的评估？ |
| 6 | 为什么股骨头坏死的分期对治疗计划很重要？ |
| **症状评估** | |
| 7 | 股骨头坏死常见哪些症状和表现？ |
| 8 | 股骨头坏死相关疼痛在什么情况下需要紧急评估？ |
| 9 | 诊断股骨头坏死通常要做哪些检查？ |
| 10 | 为什么早期股骨头坏死的X线片可能正常？ |
| 11 | MRI在股骨头坏死中能显示什么？ |
| 12 | 医生会怎样判断股骨头坏死是否已发生塌陷或关节炎？ |
| **治疗选择** | |
| 13 | 股骨头坏死有哪些治疗选择？ |
| 14 | 股骨头坏死的分期会怎样影响治疗选择？ |
| 15 | 什么是用于治疗股骨头坏死的髓芯减压术？ |
| 16 | 在什么情况下会考虑为股骨头坏死进行全髋关节置换？ |
| 17 | 对于股骨头坏死引起的疼痛，我应了解哪些药物信息？ |
| 18 | 我应怎样比较不同股骨头坏死治疗方案的获益和风险？ |
| **日常管理** | |
| 19 | 患有股骨头坏死时，我应怎样调整负重活动？ |
| 20 | 股骨头坏死患者通常适合做哪些运动？ |
| 21 | 手杖、拐杖或其他助行器能帮助减轻股骨头坏死吗？ |
| 22 | 如果我患有股骨头坏死，应该停止使用糖皮质激素吗？ |
| 23 | 患有股骨头坏死时，应怎样讨论饮酒问题？ |
| 24 | 当股骨头坏死限制髋部活动时，我应该怎样日常生活？ |
| **手术照护** | |
| 25 | 股骨头坏死接受髓芯减压术后通常需要哪些随访？ |
| 26 | 因股骨头坏死接受全髋关节置换后，我应讨论哪些康复里程碑？ |
| 27 | 在因股骨头坏死接受手术前，我应为哪些并发症做心理准备？ |
| 28 | 股骨头坏死治疗后，我何时可以恢复工作、驾驶或运动？ |
| 29 | 因股骨头坏死手术后，哪些症状需要紧急就医？ |
| 30 | 如果目前不做手术，我应该怎么监测股骨头坏死病情？ |
| **长期规划** | |
| 31 | 股骨头坏死不治疗会自行痊愈或逆转吗？ |
| 32 | 哪些因素会影响股骨头坏死进展或塌陷的可能性？ |
| 33 | 股骨头坏死会怎样影响我的情绪、睡眠或日常生活？ |
| 34 | 为什么共同决策对股骨头坏死很重要？ |
| 35 | 当我为股骨头坏死而就诊时，我应准备好哪些信息？ |
| 36 | 我怎样与医疗团队共同制定股骨头坏死的长期计划？ |
