# Supplementary material for: Cross-linguistic differences in responses to patient queries on osteonecrosis of the femoral head between English and Chinese-oriented large language models: a semantically matched bilingual cross-sectional study
Source: Front Public Health. 2026 Oct 28;14:1985180. doi: 10.3389/fpubh.2026.1985180 (PMC13752189; doi:10.3389/fpubh.2026.1985180)
Supplement: Supplementary file 3 [file Table_3.docx]

**Supplementary Table S3. English and Chinese readability measures for large language model responses to patient questions about Osteonecrosis of the Femoral Head**

| **Measure** | **Full name** | **Formula / concise description** |
| --- | --- | --- |
| **English-language readability** | | |
| FRES | Flesch Reading Ease | FRES = 206.835 − 1.015 × (words/sentences) − 84.6 × (syllables/words). |
| FKGL | Flesch–Kincaid Grade Level | FKGL = 0.39 × (words/sentences) + 11.8 × (syllables/words) − 15.59. |
| SMOG | Simple Measure of Gobbledygook | SMOG = 1.043 × √[30 × (polysyllabic words/sentences)] + 3.1291. |
| GFI | Gunning Fog Index | GFI = 0.4 × [(words/sentences) + 100 × (complex words/words)]. |
| **Chinese-language readability** | | |
| CLDC | Chinese Language Difficulty Coefficient | CLDC = ASL + ANDW |
| ARC | AlphaReadabilityChinese | Nine independent indices: lexical richness; syntactic richness; semantic accuracy for nouns, verbs, noun–verb pairs, and conjunctions; and noun semantic richness, clarity, and noise. |
| CRP | Chinese Readability Platform | Outputs a reading-difficulty score, education stage, and recommended age; only the reading-difficulty score is used for graphical analysis. |

**Abbreviations:** *ASL*, average sentence length (words per sentence); *ANDW*, average number of difficult words per 100 words.
